# FIND: A new software tool and development platform for enhanced multicolor flow analysis

**DOI:** 10.1186/1471-2105-12-145

**Published:** 2011-05-10

**Authors:** Shareef M Dabdoub, William C Ray, Sheryl S Justice

**Affiliations:** 1 Biophysics Program, The Ohio State University, 2031 Physics Research Building, 191 W. Woodruff Ave., Columbus, Ohio, USA; 2 The Battelle Center for Mathematical Medicine, The Research Institute at Nationwide Children's Hospital, 700 Childrens Dr., Columbus, Ohio, USA; 3 Center for Microbial Pathogenesis, The Research Institute at Nationwide Children's Hospital, 700 Childrens Dr., Columbus, Ohio, USA

## Abstract

**Background:**

Flow Cytometry is a process by which cells, and other microscopic particles, can be identified, counted, and sorted mechanically through the use of hydrodynamic pressure and laser-activated fluorescence labeling. As immunostained cells pass individually through the flow chamber of the instrument, laser pulses cause fluorescence emissions that are recorded digitally for later analysis as multidimensional vectors. Current, widely adopted analysis software limits users to manual separation of events based on viewing two or three simultaneous dimensions. While this may be adequate for experiments using four or fewer colors, advances have lead to laser flow cytometers capable of recording 20 different colors simultaneously. In addition, mass-spectrometry based machines capable of recording at least 100 separate channels are being developed. Analysis of such high-dimensional data by visual exploration alone can be error-prone and susceptible to unnecessary bias. Fortunately, the field of Data Mining provides many tools for automated group classification of multi-dimensional data, and many algorithms have been adapted or created for flow cytometry. However, the majority of this research has not been made available to users through analysis software packages and, as such, are not in wide use.

**Results:**

We have developed a new software application for analysis of multi-color flow cytometry data. The main goals of this effort were to provide a user-friendly tool for automated gating (classification) of multi-color data as well as a platform for development and dissemination of new analysis tools. With this software, users can easily load single or multiple data sets, perform automated event classification, and graphically compare results within and between experiments. We also make available a simple plugin system that enables researchers to implement and share their data analysis and classification/population discovery algorithms.

**Conclusions:**

The **FIND **(Flow Investigation using N-Dimensions) platform presented here provides a powerful, user-friendly environment for analysis of Flow Cytometry data as well as providing a common platform for implementation and distribution of new automated analysis techniques to users around the world.

## Background

The advent of Flow Cytometry (FC) and subsequent enhancements allowing for polychromatic investigation has proved invaluable to those involved in basic research and medical diagnosis. By differentially staining the cell population using dyes specific for various characteristics, each cell produces a fluorescence signature characteristic of the bound dyes. Thus, by measuring the fluorescence at multiple wavelengths, a multidimensional quantitative signature can be defined for each cell. However, from the time of introduction the overwhelmingly predominant method for analyzing and interpreting data gathered from flow cytometers has been manual gating. In this process, event data is visualized using bivariate plots such as histograms, contour, scatter, and density plots. Users must then peruse the entirety of the data, two channels (dimensions) at a time and manually isolate sections of the plot by visually applying geometric constructs (rectangles, circles, ellipses) to group or separate the data. In this manner, specific cell populations are identified and separated from the mass of data.

With advances in multi-laser flow cytometers, commercial machines are now capable of gathering up to 20 channels (or more using quantum dots over traditional organic fluorophores [1]) of data per event. In addition, current research into adding mass spectrometry capability to flow cytometers (mass cytometry [2]) promises determination of up to 100 different biomarkers. The number of unique 2D plots needed to cover a dataset is , thus for 20 channels of data almost 190 plots are necessary, and for 100 channels, nearly 5000 plots.

**Figure 1:**
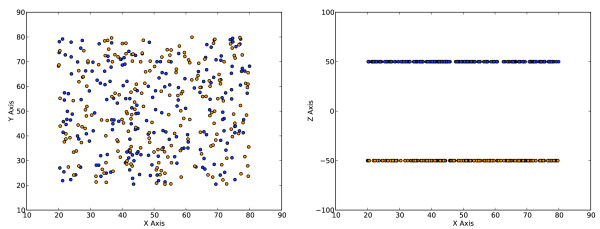
**An illustration of the difficulty analyzing multi-dimensional data given only 2D views**. (**a**) A 2D slice of a 3D data set gives the impression of uniformity. (**b**) Switching out the second dimension for the third dimension clearly shows this to be a false assumption.

Beyond the great deal of time needed to process such a large number of graphs, it is impossible (in the general case) for two-dimensional slices to give an accurate picture of the distribution of points in N-dimensional space (Figure 1). Furthermore, segmentation using canonically aligned 2D planes in an N-dimensional data space can only correctly isolate a special subset of features where the correct segregation is aligned with the axes. For these reasons, applying the techniques of multivariate data classification (from the fields of mathematics and computer science) has been the subject of research for the last 20 years [3-6]. Unfortunately, the published body of research into automating analysis has been spotty over this time period [7]. Even more disappointing is the fact that the vast majority of this research has never made widespread appearance in analysis software for the end user, thus remaining simply research. It seems clear however, that realizing the true usefulness of flow cytometry is dependent on the development of automated multidimensional classification techniques [7,8].

The main barrier to the widespread adoption of such tools is a technological one. Published research describing classification methods takes one of two routes. Authors may write their own processing code and rely on importing analyzed data into generic or FC-specific programs for visualization and data-interaction [3,5,6]. Alternately, some authors have repurposed software intended for analysis of other types of data such as microarrays [9]. While ultimately accomplishing their purpose, such methods are far from ideal for reusability and wider adoption. Indeed, these approaches expose an underlying need: a user-friendly, purpose-built software platform on which researchers can easily implement analysis techniques, algorithms, and new visualizations. In this paper, we present the development of such a platform with the main goal of easing future development and encouraging widespread use of alternate tools, providing a system that makes complete use of the available data dimensions. Consequently, our tool will greatly aid user analysis in time as well as consistency.

### Existing Software

The main analytical focus of the most widely used industry software, such as FlowJo and FCS Express, is manual gating. FlowJo in particular provides one method of algorithmic analysis, based on Probability Binning [10], however it is currently in beta status and available on only one platform. Such software with large user bases are understandably slow to change, and perhaps reluctant to provide users with analysis tools that are not widely recognized.

Currently available software providing automated analysis methods include the more generalized analysis packages such as the flow cytometry suite [11], provided as part of the Bioconductor [12] project, the stand-alone software Flow [13], and specialized tools such as that provided by the authors of the recently published automated analysis technique: FLAME [14].

The FC analysis libraries available through the Bioconductor project are excellent, yet, while being based on R has many benefits, it is at its core a command-line based software. For many users, command-line access is a foreign concept, and this fact alone will prevent them from ever trying it. However, far from being in competition with Bioconductor, there is potential for FIND to make use of Bioconductor through a bridge library, RPy, that allows access to R functionality from Python programs. The program Flow suffers from similar problems in the area of usability. The interface itself can be confusing and tends to be overly technical, as is the documentation which states the program is aimed more at developers than end-users. Software in the category of algorithm-specific programs, such as the webservice FLAME, certainly serve a useful purpose, but offer no generality or measures for comparison to other methods. No algorithm will be appropriate for every dataset, and continuity of interface is important for work-flow efficiency.

## Implementation

The Python programming language was chosen for its high-level syntax, ease of use, multi-platform independence, and the availability of excellent scientific and numerical analysis packages (among them, SciPy and NumPy). In addition, Python interfaces well with the C++ and Java programming languages, allowing code in both to be used from within Python programs (Boost.Python and Jython, respectively). Our foremost goals while developing FIND were: user friendliness, and simplicity of design, implementation, and maintenance. In pursuit of these goals, we chose the wxWidgets library which allows true cross-platform development of windowed applications with the native look and feel of the operating system under which the program is run.

The design and implementation of FIND was guided by the well-known Model-View-Controller (MVC) architectural pattern, in which the underlying data model is separated from the user interface and the controlling system logic. This design paradigm allows for greater flexibility, clean implementation, and simplified overall debugging and maintenance. The other major concerns in design centered around the two user populations we envisioned for this software: Normal users of Flow Cytometry analysis software, and researchers involved in improving the analysis and visualization of FC data. The former target population was represented throughout the development process by five researchers experienced in the design and analysis of FC experiments. The latter population was considered carefully throughout and well-represented by the developer.

Finally, we provide pre-built platform specific executables for Microsoft Windows and Mac OS X. These packages are entirely self-contained and require no additional software to be installed. Additionally, all plugins are located in a folder external to the executables, allowing users to easily "install" extended functionality to the program.

## Results and Discussion

The first concern in the design of FIND was simplifying the user experience and streamlining the analysis process. Thus, FIND consists of a single window split into a data pane and a display pane. The data pane lists loaded data sets, their subdivisions and clusterings, as well as stored figure sets, in a hierarchical tree. Each item in the tree is selectable and has a context menu available with a number of generic and item-specific actions available to the user. For example, all items may be plotted, but plotting methods are specified as applicable to data items alone, clustering items alone, or both.

The display pane is further subdivided into a plotting pane and a dimension-selection pane. The plotting pane may be configured to display a grid of subplots, each selectable and configurable independently. This forms the heart of the display system and was designed to collect data visualizations in one location. By default, all subplots are bound to the user-selected dimensions, and are updated instantly when the selection is changed. Individual plots may be unbound via a simple checkbox that indicates the bound status of the currently selected subplot.

**Figure 2:**
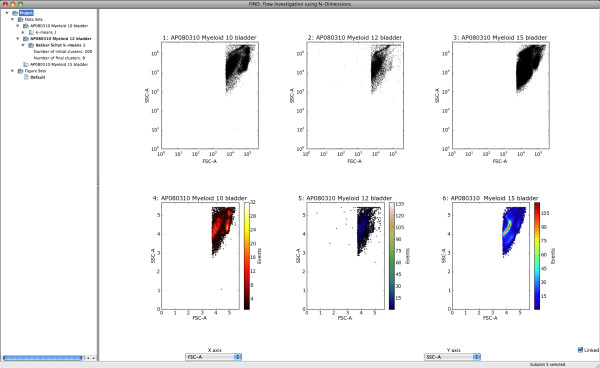
**A snapshot of the basic visual elements of FIND running under OS X Leopard**. The data pane to the left contains all loaded datasets, any subsets or analyses belonging to each, and a list of stored Figures. The plotting pane on the right displays in grid form any data visualizations. The dimension selection pane, below the plotting pane, allows users to select which dimensions are plotted in the above pane. Upon selection, any updatable plots linked to the dimension selectors are replotted with the new dimensions.

In order to ease the issue of commands, at all times the system tracks four "selected items": the currently selected data set, clustering, figure set, and subplot (assuming at least one of each exists). The selected items in the tree view of the data pane are highlighted by bold text, and the selected subplot is indicated in the status-bar at the bottom of the application (Figure 2). Menu options apply to the appropriate selected item, reducing as much as possible the amount of effort required by the user to translate thought into result.

### Data Analysis

**Figure 3:**
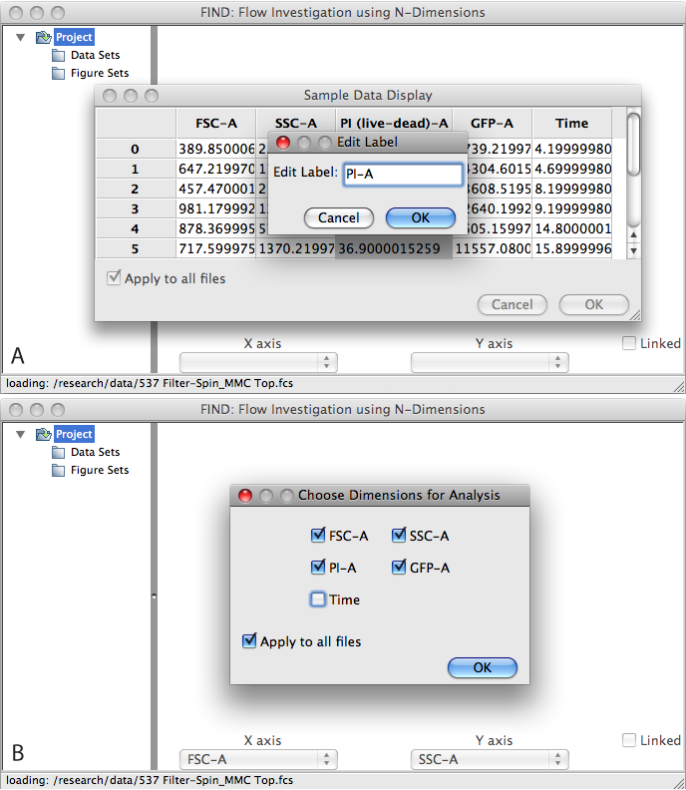
**A screenshot of FIND opening an FCS file**. (**a**) The sample data display dialog allows users to rename and rearrange columns. (**b**) The user can select which dimensions are used for automated analysis algorithms.

A typical workflow begins with the user opening one or more related data files for analysis. These files may be FCS 3.0 standard files, CSV files, or any additional file types that a plugin might be written for. Each file is parsed and a display of the first ten rows of data is presented, giving the user a preview of the data as well as an opportunity to alter any of the column labels or their arrangement (Figure 3a). Having arranged or renamed to their satisfaction, the user can exclude columns of data (such as Time) from automated analysis procedures (Figure 3b). The files are loaded and data and any annotations extracted (e.g. the TEXT segment in FCS-formatted files). Each data set is plotted as a 2D scatterplot in a 2 × N grid with the selected dimensions set to the Forward and Side-scatter channels.

**Figure 4:**
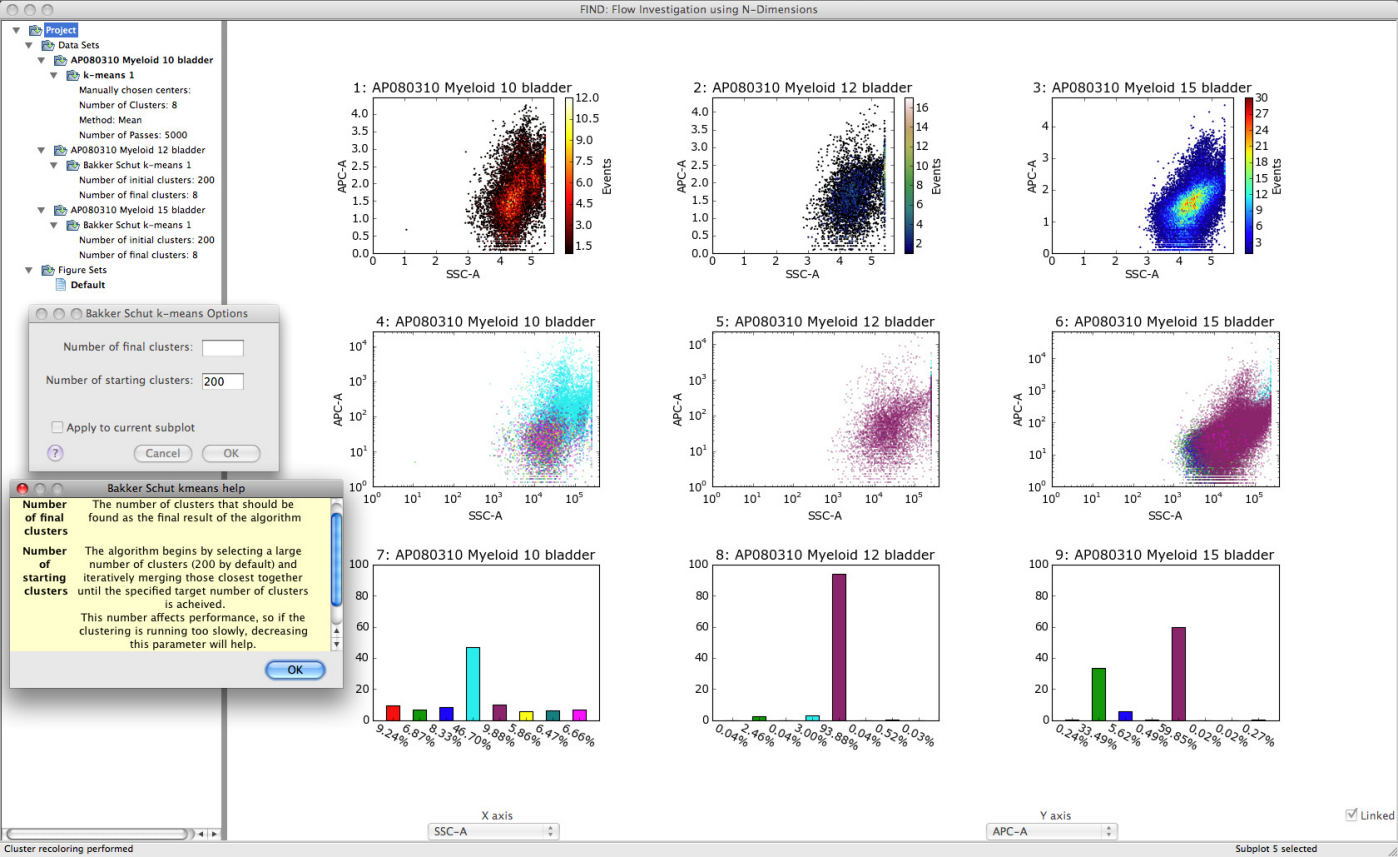
**Using FIND to perform automated cluster analysis on three data sets**. Row 1: Scatter plots of the original data. Row 2: The Bakker Schut *et al. *[3] modified k-means algorithm is applied to two data sets and the resulting clusters are visualized with 2D scatterplots coded by color. The overlay features an example of an options dialog (top) for a clustering algorithm as well as the provided inline help system (bottom).

**Figure 5:**
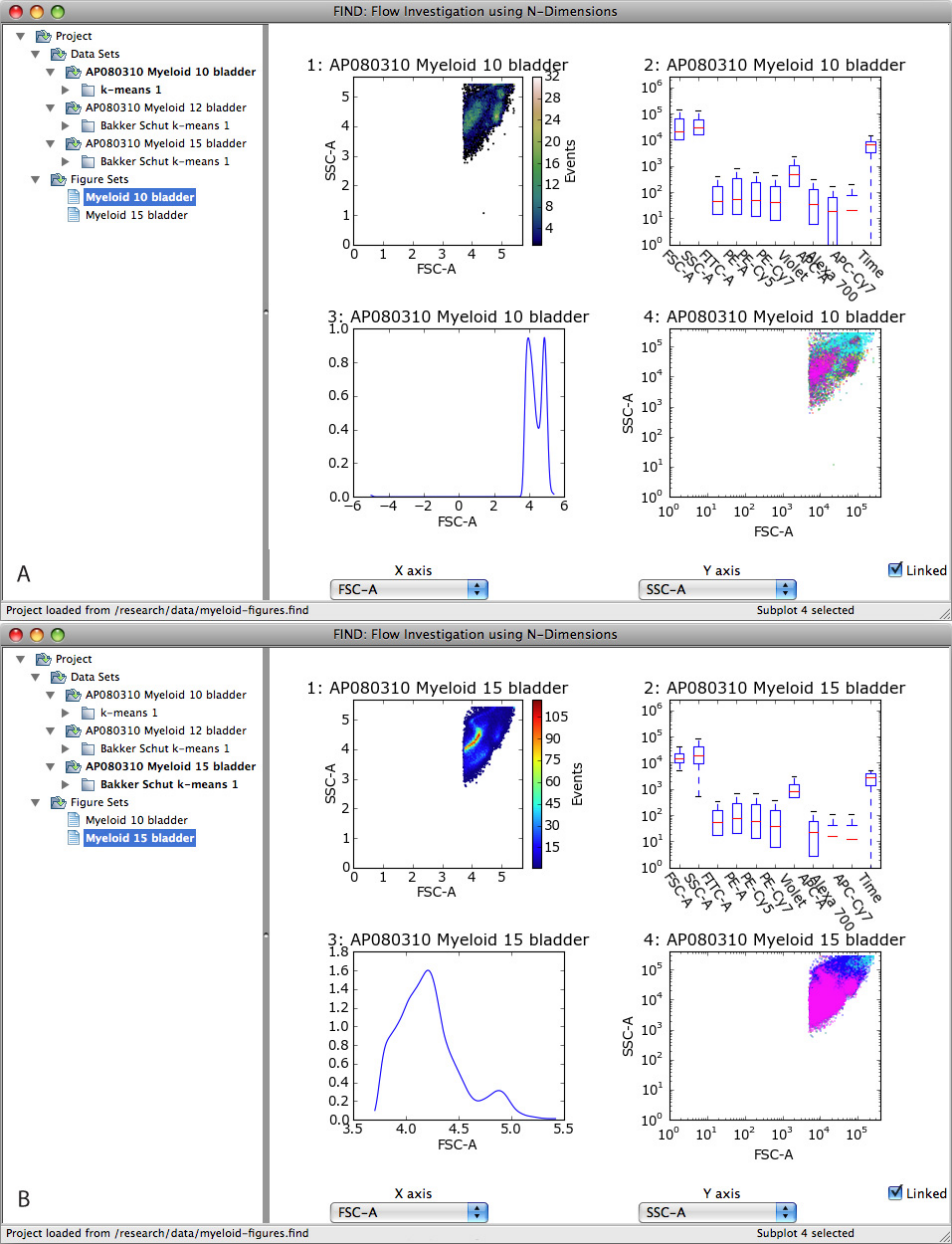
**An illustration of Figure Sets**. Figure Sets provide the means to create any number of plot groupings. Here we have used two Figures to separately display visual summaries of the available data.

Before performing automated analysis, the user may wish to begin the analysis with a visual data exploration. Currently FIND provides the following 2D plots: scatterplots, histograms, heatmaps, and side-by-side boxplots of all dimensions selected for analysis. With the simple configurable grid plot structure of the display pane, any or all of these plots can be displayed together for one or more of the loaded data sets (Figure 4). Additionally, each plot has its own properties dialog, allowing the user to configure aspects of the plot such as range, data transformation (linear, log, etc...), and other plot-specific options. Due to the limiting factor of screen space, FIND enables users to create groupings of plots, called Figures. Each Figure, represented in the data pane (Figure 2), stores the contents of the display pane such that clicking on a Figure Set entry switches the display to the plots and selections within that Figure. This gives users great freedom to create more plots than would ordinarily be possible, as well as, for example, to focus analysis of different populations to individual Figures (See Figure 5).

**Figure 6:**
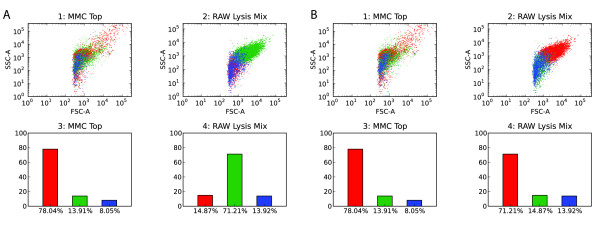
**Cluster Reordering**. Due to non-deterministic components of many clustering algorithms, similar clusters are not always found in the same relative order, thus making visual comparison more difficult. Here we have used a measure of cluster similarity [3] to reassign cluster colorings, making such analysis easier. (**a**) and (**b**) Before and after recoloring, respectively. Both images are figures exported from within FIND.

Continuing the analysis, FIND enables users to perform automated population discovery, also known as cluster analysis or automatic classification. FIND currently includes an implementation of the algorithm created by Bakker Schut *et al. *[3]. Briefly, the user initially specifies a target number of clusters for discovery. The algorithm performs a log transformation and chooses a high number of nonrandom [15] initial centers and uses these seeds as input to the traditional k-means clustering algorithm. The resulting clusters are then iteratively merged together using statistical cluster shape comparison (also described by Bakker Schut *et al. *[3]) to discover those most similar, until the user-specified target is achieved (for algorithm analysis and results see [3]). The resulting clusters (if desired by the user) are then plotted to the current subplot in a scatterplot overlay color-coded by cluster (Figure 4). Statistical information on the clusters is available as a context menu item when clicking on the desired tree item in the data pane. A bar chart visualizing the overall event percentages in each cluster is also available (Figure 6).

One consequence of many automatic classification algorithms is some degree of non-determinism in cluster ordering. This does not negatively impact the resulting analysis, but it does pose an issue for later visualization. The simplest method for visually distinguishing the events belonging to different clusters is through color variation. However, for such differentiation to be more meaningful across multiple runs of an algorithm, whether for the same or separate data sets, similar clusters should be given the same color. Thus, we have applied the measure of cluster similarity used in the Bakker Schut *et al*. algorithm [3] to reorder the clusters such that those most similar appear with the same color when plotted (Figure 6).

**Figure 7:**
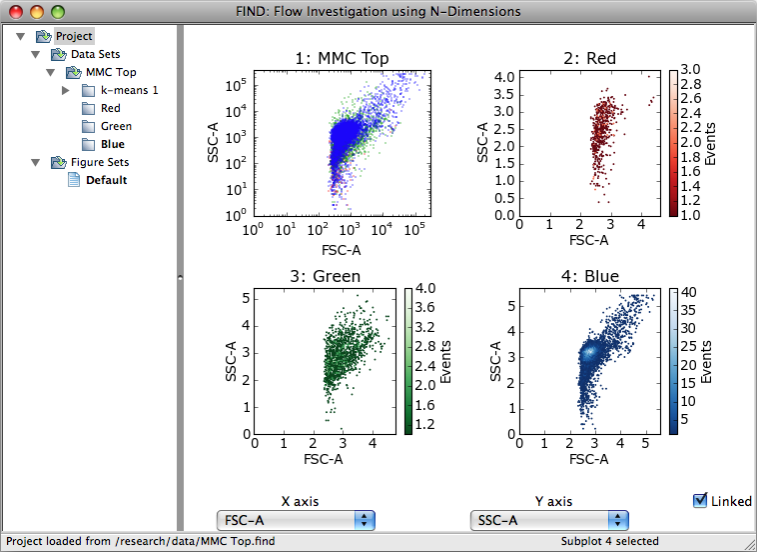
**Cluster Isolation**. Using FIND to isolate one or more clusters and create new datasets as children of the original parent dataset.

FIND allows for the isolation and extraction of individual, or combinations of clusters for further analysis. When viewing the clustering summary (Figure 7), any combination of clusters may be selected and copied to a new dataset. These new data sets are attached, as children, to the parent data set from which they were extracted. Child datasets can be accessed, analyzed, and visualized using all the means available for one opened from a file, but appear in the data tree as children of the datasets from which they were isolated. A group of child datasets can be created for each discovered cluster, allowing for independent visualization and analysis of each. In this manner, users are enabled to explore as many levels of their data as they desire. Additionally, users may wish to perform analysis or visualization with other programs, and FIND provides the option to export any dataset to an external file type supported by FIND or available plugins.

### Analysis Completion

While the grid layout display provided by FIND enables easy comparative visualization and analysis of multiple datasets, it doubles as a figure creation tool. Users simply select the 'Save Figure' menu item in the Plot menu, and are able to instantly create usable figures for presentation or publication in the following formats: PNG, PDF, PostScript, EPS (Encapsulated PostScript), and SVG (Scalable Vector Graphics). Exporting the current visualization grid eliminates the need for additional steps in figure creation and additional complexity in subplot placement, thus saving time and effort (Figure 6).

One of the most important steps in analysis at any point, and especially the end, is saving the work that has been done. FIND allows users to save the entire state of the analysis as two files: one containing all the qualitative information about data structure, attributes, and visualizations, the second a simple binary file containing all the loaded numeric data. These two files can be packaged and transported in any manner for later use on the same machine or any other machine running FIND. Finally, all of the preceding topics are discussed in more detail and with examples in the user documentation included in Additional file 1 as well as on the project website.

### Plugin System

A major component of the FIND application is enabling researchers and software developers to extend the functionality of the system in various ways. The main focus of the plugin system is automated population discovery and giving researchers a common platform for implementing their research. However, FIND provides additional access modalities; specifically we have categorized them into the following: Graphing, Transformations, I/O, and Analysis.

Graphing plugins offer authors a hook into the plotting subsystem to implement new visualization options. In order to reduce complexity, graphing plugins are visible in the Plugins menu (indicating their availability), but only usable from the plot submenu in the data tree context menu. Furthermore, authors specify graphing plugins as applicable to either datasets (items in the tree representing a file or a child dataset) or clusterings. For example, the scatterplot is applicable to both, while the histogram plot is only available for datasets. As a result, the context menu is built dynamically to fit the tree item selected. Transformation plugins provide means to transform the data to a more meaningful data space, such as the widely used Hyperlog [16] transformation. All transformations (plugin or built-in) can be called through the FIND API and applied to given datasets. I/O (input/output) plugins allow the import and export of data and automated analysis results. This will enable the inclusion of various data formats from other programs/FC machines, and allow for FIND results to be saved for later use as needed. The last modality, Analysis, is intended to provide an all-purpose means for plugin authors to offer novel analysis or visualization options that do not fall into any of the other plugin categories. On the user side, plugins are easily installed by saving the plugin files into the appropriate subdirectory of the plugins directory located with the FIND executable. Complete details on writing plugins for FIND, with code examples, are available in the developer documentation included in Additional file 1 as well as on the project website.

Finally, as software engineering is never a flawless science, errors and bugs will occur. Thus FIND catches unexpected errors, and can report these directly to us. This will aid in the development and improvement of FIND, as well as provide users a more technically specific means of feedback. In addition, FIND provides a mechanism enabling users to check for updates to the program, allowing them to stay current with fixes and enhancements as development continues.

## Conclusions

It is becoming clear that the advance of biochemical technology will soon outstrip the ability of users to manually analyze Flow Cytometry data effectively and in a reasonable amount of time. Two-dimensional visual analysis is useful, but at the very least should be combined with multi-dimensional mathematical analysis to minimize the risk of losing important information. The answer lies in the direction of intelligent automated analysis tools [7], however, without a user-friendly platform on which to implement, widespread adoption of such tools has been and will continue to be extremely difficult. Additionally, it is wasted and redundant effort for researchers to reinvent the wheel or attempt to repurpose existing software each time a new analysis tool is developed.

A more serious problem lies in the fact that, without widespread testing of algorithms on multiple different datasets, it will be difficult to verify the accuracy and usefulness of new software analysis tools. Fortunately, these needs are coming to be recognized in the form of calls for increased development and improvement [7,8], the IEEE Bioinformatics Standards for Flow Cytometry Working Group designed to provide data standard proposals, and an assessment competition (FlowCAP) similar to the annual protein structure prediction competition CASP. FIND can be very useful in facilitating these efforts, providing a platform upon which to build and test.

Finally, it should not be overlooked that automated analysis methods which simply present the researcher with results as a finished product, but which do not facilitate the researcher's comprehension by supporting exploration and evaluation of the results, are almost as non-productive as a total lack of automation. It has been repeatedly demonstrated that human experts either reject and manually re-do automated analyses which are not presented in such a way as to facilitate exploration, comparison and understanding, or come to rely upon these analyses without adequate concern for confirming their ongoing validity [17-20]. Even the best-of-breed research algorithms for FC analysis therefore fail to deliver the benefits that they could, because they universally operate in isolation, and do not provide internal comparisons to other methods. Such omissions, as trivial as they may seem, critically impede the wide adoption of improved methods, and absorb considerable human resources which could be released for more productive tasks if the improved methods can be incorporated within a familiar comprehensive analysis framework.

To conclude, here we have presented FIND, a new cross-platform software package that provides a basic visualization and development platform for analysis of Flow Cytometry data, while maintaining a focus on end-user accessibility. FIND presents an easy to use integrated interface, behind which exists a powerful plugin system based on the modern, widely used language Python, and the excellent numeric and scientific computational toolset available to it.

## Availability and requirements

**Project name**: Flow Investigation using N-Dimensions

**Project home page**: http://www.justicelab.org/find

**Alternate page**: http://www.mathmed.org/#find

**Operating Systems**: Platform independent

**Programming language**: Python

**Other requirements**: None

**License**: Version 0.3.1 of FIND is released under the GPLv3

**Restrictions**: FIND is free for Academic use only

## Authors' contributions

SMD conceived, designed, and implemented the software, and drafted the manuscript. WCR and SSJ supervised and guided the design and implementation, and helped to draft the manuscript. All authors read and approved the final manuscript.

## Links

**FlowJo **http://www.flowjo.com

**FCS Express **http://www.denovosoftware.com

**Massively Multiparametric Mass Cytometer Analyzer Project **http://www.stemspec.ca

**FLAME: FLow analysis with Automated Multivariate Estimation **http://www.broadinstitute.org/cancer/software/genepattern/modules/FLAME

**RPy **http://rpy.sourceforge.net

**SciPy and NumPy **http://www.scipy.org

**Boost.Python **http://www.boost.org/doc/libs/1_45_0/libs/python/doc/index.html

**Jython **http://www.jython.org

**wxWidgets **http://www.wxwidgets.org

**wxPython **http://www.wxpython.org

**Bioinformatics Standards for Flow Cytometry **http://flowcyt.sourceforge.net

**FlowCAP - Flow Cytometry: Critical Assessment of Population Identification Methods **http://flowcap.flowsite.org

**Critical Assessment of protein Structure Prediction (CASP) **http://predictioncenter.org

## Supplementary Material

Additional file 1 **FIND User and Developer Documentation**. This document provides a complete introduction and tutorial for the FIND end-user as well as a complete description, tutorial, and code examples on developing plugins for the FIND platform. This material is also available online at the project website. Click here for file

## References

[B1] Perfetto SP Chattopadhyay PK Roederer M Seventeen-colour flow cytometry: unravelling the immune system Nat Rev Immunol 2004 4 8 648 655 15286731 10.1038/nri1416

[B2] Bandura DR Baranov VI Ornatsky OI Antonov A Kinach R Lou X Pavlov S Vorobiev S Dick JE Tanner SD Mass Cytometry: Technique for Real Time Single Cell Multitarget Immunoassay Based on Inductively Coupled Plasma Time-of-Flight Mass Spectrometry Analytical Chemistry 2009 81 16 6813 6822 19601617 10.1021/ac901049w

[B3] Schut TCB Grooth BGD Greve J Cluster analysis of flow cytometric list mode data on a personal computer Cytometry 1993 14 6 649 659 8404371 10.1002/cyto.990140609

[B4] Wilkins MF Hardy SA Boddy L Morris CW Comparison of five clustering algorithms to classify phytoplankton from flow cytometry data Cytometry 2001 44 3 210 217 11429771 10.1002/1097-0320(20010701)44:3<210::aid-cyto1113>3.0.co;2-y

[B5] Lugli E Pinti M Nasi M Troiano L Ferraresi R Mussi C Salvioli G Patsekin V Robinson JP Durante C Cocchi M Cossarizza A Subject classification obtained by cluster analysis and principal component analysis applied to flow cytometric data Cytometry Part A 2007 71A 5 334 344 10.1002/cyto.a.20387 17352421

[B6] Finn WG Carter KM Raich R Stoolman LM Hero AO Analysis of clinical flow cytometric immunophenotyping data by clustering on statistical manifolds: Treating flow cytometry data as high-dimensional objects Cytometry Part B: Clinical Cytometry 2009 76B 1 7 10.1002/cyto.b.20435 18642311

[B7] Lizard G Flow cytometry analyses and bioinformatics: Interest in new softwares to optimize novel technologies and to favor the emergence of innovative concepts in cell research Cytometry Part A 2007 71A 9 646 647 10.1002/cyto.a.20444 17680704

[B8] Lo K Brinkman RR Gottardo R Automated gating of flow cytometry data via robust model-based clustering Cytometry Part A 2008 73A 4 321 332 10.1002/cyto.a.20531 18307272

[B9] Hofmann M Zerwes H Identification of organspecific T cell populations by analysis of multiparameter flow cytometry data using DNA-chip analysis software Cytometry Part A 2006 69A 6 533 540 10.1002/cyto.a.20278 16646049

[B10] Roederer M Treister A Moore W Herzenberg LA Probability binning comparison: A metric for quantitating univariate distribution differences Cytometry 2001 45 37 46 11598945 10.1002/1097-0320(20010901)45:1<37::aid-cyto1142>3.0.co;2-e

[B11] Hahne F LeMeur N Brinkman RR Ellis B Haaland P Sarkar D Spidlen J Strain E Gentleman R flowCore: a Bioconductor package for high throughput flow cytometry BMC Bioinformatics 2009 10 106 [PMID: 19358741] 19358741 10.1186/1471-2105-10-106 PMC2684747

[B12] Gentleman RC Carey VJ Bates DM Bolstad B Dettling M Dudoit S Ellis B Gautier L Ge Y Gentry J Hornik K Hothorn T Huber W Iacus S Irizarry R Leisch F Li C Maechler M Rossini AJ Sawitzki G Smith C Smyth G Tierney L Yang JYH Zhang J Bioconductor: open software development for computational biology and bioinformatics Genome Biology 2004 5 10 R80 [PMID: 15461798] 15461798 10.1186/gb-2004-5-10-r80 PMC545600

[B13] Frelinger J Kepler T Chan C Flow: Statistics, visualization and informatics for flow cytometry Source Code for Biology and Medicine 2008 3 10 18559108 10.1186/1751-0473-3-10 PMC2442075

[B14] Pyne S Hu X Wang K Rossin E Lin T Maier LM Baecher-Allan C McLachlan GJ Tamayo P Haer DA Jager PLD Mesirov JP Automated high-dimensional flow cytometric data analysis Proceedings of the National Academy of Sciences 2009 106 21 8519 8524 10.1073/pnas.0903028106 PMC2682540 19443687

[B15] Arthur D Vassilvitskii S k-means++: the advantages of careful seeding Proceedings of the eighteenth annual ACM-SIAM symposium on Discrete algorithms 2007 New Orleans, Louisiana: Society for Industrial and Applied Mathematics 1027 1035

[B16] Bagwell CB Hyperlog-A flexible log-like transform for negative, zero, and positive valued data Cytometry Part A 2005 64A 34 42 10.1002/cyto.a.20114 15700280

[B17] Parasuraman R Riley V Humans and automation: Use, misuse, disuse, abuse Human Factors 1997 39 2

[B18] Skitka LJ Mosier K Burdick MD Accountability and automation bias International Journal of Human-Computer Studies 2000 52 4 701 717

[B19] Wickens CD Imperfect and unreliable automation and its implications for attention allocation, information access and situation awareness 2000 Tech. rep., University of Illinois at Urbana-Champaign

[B20] Lee JD See KA Trust in Automation: Designing for Appropriate Reliance Human Factors: The Journal of the Human Factors and Ergonomics Society 2004 46 50 80 10.1518/hfes.46.1.50_30392 15151155

