# Supplementary material for: FIND: A new software tool and development platform for enhanced multicolor flow analysis
Source: BMC Bioinformatics. 2011 May 10;12:145. doi: 10.1186/1471-2105-12-145 (PMC3119067; doi:10.1186/1471-2105-12-145)
Supplement: Additional file 1 — FIND User and Developer Documentation. This document provides a complete introduction and tutorial for the FIND end-user as well as a complete description, tutorial, and code examples on developing plugins for the FIND platform. This material is also available online at the project website. [file 1471-2105-12-145-S1.PDF]

FIND Documentation

Release 0.3.1

Shareef Dabdoub

February 13, 2011

CONTENTS

1 FIND User Documentation 3
1.1 Introduction 3
1.2 Data 5
1.3 Visualization 8
1.4 Data Analysis 15

2 FIND Developer Documentation 21
2.1 Introduction 21
2.2 Analysis Plugins 22
2.3 Clustering Plugins 23
2.4 Graphing Plugins 25
2.5 Input/Output Plugins 30
2.6 Transformation Plugins 31

3 Indices and tables 33

Release 0.3

Date February 13, 2011

FIND (**F**low **I**nvestigation using **N**-**D**imensions) is a software package designed to facilitate the visualization and analysis of Flow Cytometry data for the scientific target audience,

Contents:

CHAPTER

ONE

FIND USER DOCUMENTATION

Contents:

1.1 Introduction

The FIND software platform is designed to be a user-friendly tool for analysis and visualization of Flow Cytometry data, revolving around automated methods based on multi-dimensional mathematical inspection of relationships between sampled cells and their recorded fluorescence emissions.

FIND is also designed to be a platform for development and distribution of new analysis and visualization methods, simplifying the process of finding and using new research in Flow Cytometry analysis. If you are interested in developing plugins for FIND, please visit the developer documentation on the main documentation page (link at the top left).

The main website for FIND is located at:

http://www.justicelab.org/find

The current version (as indicated at the top of the page) of FIND is available there for download, as well as any plugins. For help and questions that are not addressed here, support forums exist at FIND's Sourceforge project page.

1.1.1 User Interface

Below is a screenshot of the basic user interface for FIND. The major interface elements are highlighted, as well as the important data FIND keeps track of at all times.

1. **Project Tree** - This is a hierarchical representation of the loaded datasets and their clusterings (where existing), and the list of stored Figure Sets (see Visualization section). Much of the user interaction with these items occurs from within this tree through the context menus of individual items. This tree also provides visual clues to help the user identify the current selection for various items. These current selections, as discussed in detail below, help guide user interaction as many menu actions operate on them.
2. **Plotting Area** - This rectangular area contains all the graphs and visualizations. It is divided into a grid layout (rows and columns) that can be set by the user (menu **Plots>>Setup**). Everything contained within this area is stored in the Figure it belongs to, and selecting a different Figure from the list of

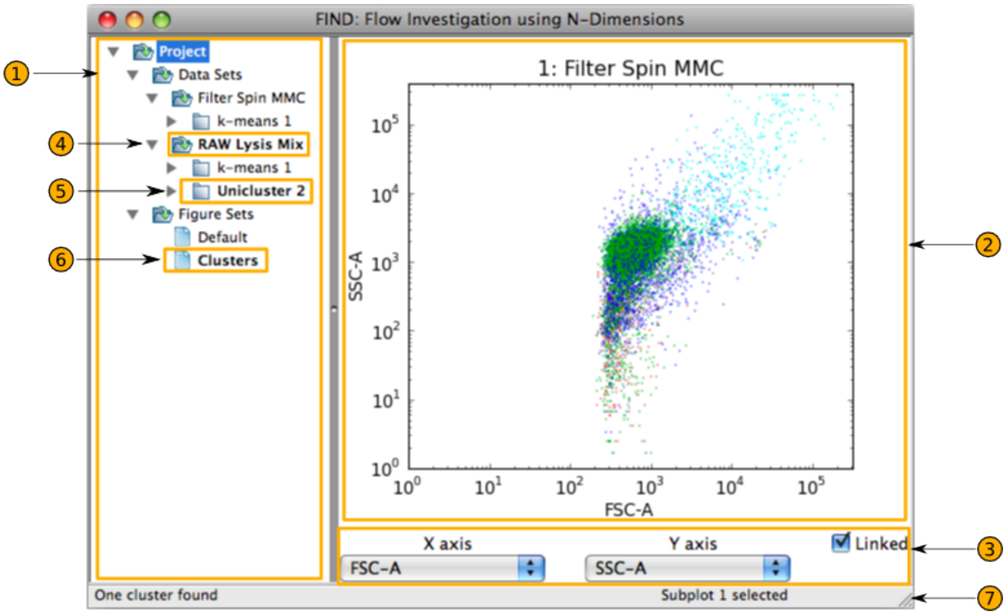

Figure Sets in the Project Tree will replace the contents of the Plotting Area with the plots stored in the newly selected Figure.

3. **Channel Selection Panel** - The dropdown boxes in this panel contain the channels (dimensions) within the loaded datasets. The selected channel, as reflected by the display within the box, controls the displayed channels in the plots that display single channels (2D Scatterplot, Histogram, etc...). When a channel is changed in one of the selectors, every plot that is affected is updated and redrawn, thus each of these are "linked" to the channel values in the selection boxes. If you want to "freeze" individual plots such that they do not respond to channel selection changes, simply click on the plot, and uncheck the box next to the "Linked" label on the right side of the panel.

4. **Currently Selected Dataset** - For all the loaded datasets, there is a single dataset that is considered the "selected" dataset. FIND uses this selection to determine which dataset to operate on when the user selects menu actions such as clustering algorithms or various plugins. Visually, as can be seen in the above image, this dataset is distinguished by a bold font.

5. **Currently Selected Clustering** - This operates in a similar fashion to the selected dataset. Currently it is only important when adding a new plot to the Plotting Area (**Plots>>Add New Subplot**). If the current dataset also has one or more clusterings, the selected clustering will be plotted as a colored 2D Scatterplot (indicating different clusters) when the new plot is created. As with the selected dataset, the selected clustering is indicated by a bold font.

6. **Currently Selected Figure** - This selection (also in bold font) indicates the Figure that is currently active and displayed in the Plotting Area. To switch the displayed Figure, you can simply click on the Figure you want displayed and FIND will automatically switch out the old Figure for the new Figure. Note that the current Figure cannot be deleted, so you must switch to a different Figure, then right-click on the Figure you want to delete, and select **Delete** from the context menu.

7. **FIND Status Bar** - The status bar is divided into two regions. The region on the right is used to display messages to the user based on actions they have performed, or short explanations of menu items the mouse is hovering over. The left side of the bar is used to indicate the final Selected Item that FIND keeps track of: the Currently Selected Plot. The selection is made by left-clicking on an

individual plot (subplot) within the Plotting Area. This allows FIND to determine where new plots should be drawn to when plotting a dataset or clustering through the context menu for those items.

1.1.2 Updating FIND

You can check for updates to FIND through the **Help** menu. If an update is available, FIND will notify you and offer to open the website so you can download the newest version.

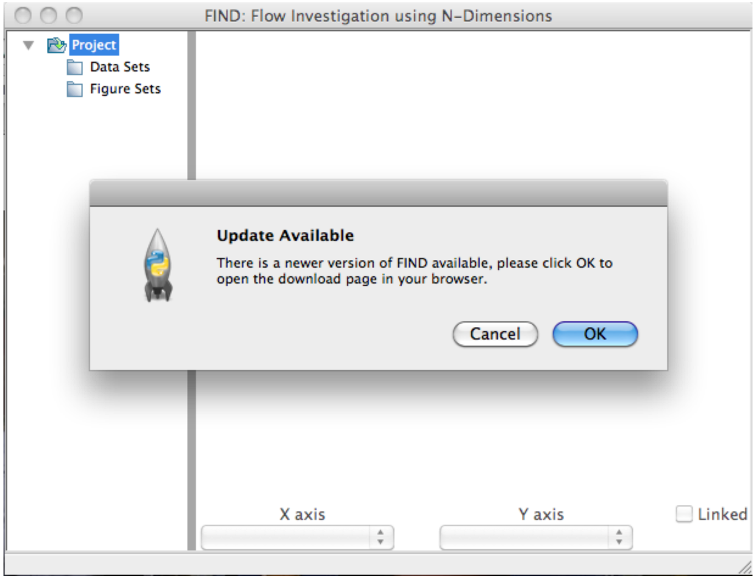

1.2 Data

Currently, the main source FIND accepts for data are FCS 3.0 formatted data files. These files contain a great deal of useful information that FIND can make use of to provide a better user experience. However, FIND can also open CSV (comma separated value) files in the following format:

```
"FSC-A","SSC-A","PI (live-dead)-A","GFP-A"
389.850006103516,2.46000003814697,-14.7600002288818,1739.21997070312
.
.
.
```

Additional file formats may be used as a source if a plugin is available to handle them.

1.2.1 Opening files

Flow Cytometry (FC) data files are opened through the File >> Open... menu item in the FIND interface. As mentioned in the previous section, the main data source are FCS 3.0 files, and those are enabled for selection by default. The screenshots below illustrate the process of opening single or multiple files.

Open File dialog with FCS 3.0 selected by default

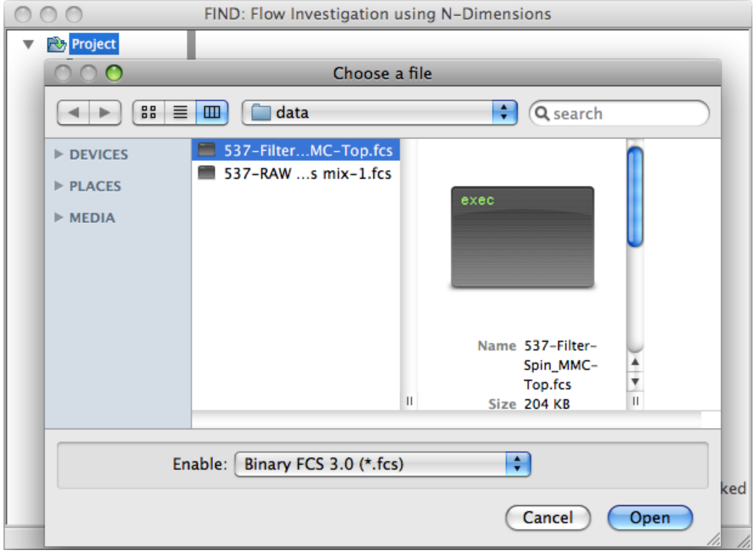
FIND: Flow Investigation using N-Dimensions
Project
Choose a file
data
search
DEVICES
PLACES
MEDIA
537-Filter...MC-Top.fcs
537-RAW ...s mix-1.fcs
exec
Name 537-Filter-Spin_MMC-Top.fcs
Size 204 KB
Enable: Binary FCS 3.0 (*.fcs)
Cancel
Open

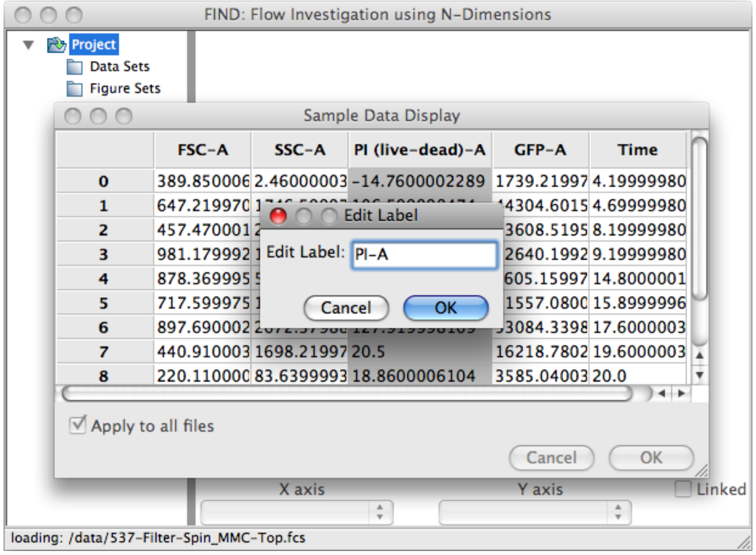
FIND: Flow Investigation using N-Dimensions
Project
Data Sets
Figure Sets
Sample Data Display
FSC-A
SSC-A
PI (live-dead)-A
GFP-A
Time
Edit Label
Edit Label: PI-A
Cancel
OK
Apply to all files
Cancel
OK
X axis
Y axis
Linked
loading: /data/537-Filter-Spin_MMC-Top.fcs

After choosing your file(s) the Sample Data Display window appears with all the channels and the first ten rows of data visible. The data grid allows you to rearrange columns by clicking on the column header and dragging to the desired position. Double clicking on the column header allows you to rename the channel, as illustrated above. If you are opening multiple files with the same column order, check the 'Apply to all files' option. This will apply the column order and names to all following files.

Note: When opening multiple files, FIND assumes each experiment represented by the files was done with the same channels, and will use the channel names from the first opened file to populate the Dimension Selection dropdowns.

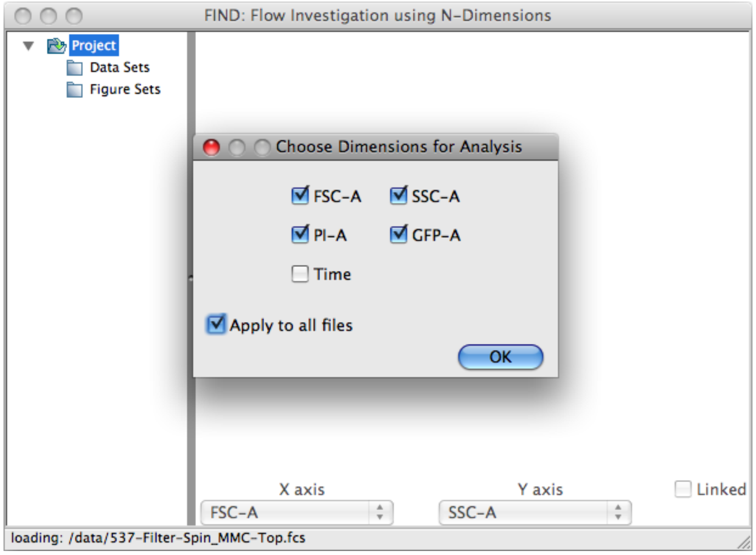

The second dialog displayed when opening FC data files allows you to specify which channels are used when applying automated analysis tools such as clustering algorithms. For example, in many cases the automatically recorded Time channel does not provide useful information for classifying cells. In the screenshot above, it has been deselected. Again, by selecting 'Apply to all files' the selections made here will automatically be applied to the rest of the files being opened.

The two screenshots below illustrate the result of opening, respectively, one and multiple files. FIND automatically plots a 2D scatter plot of the first two channels for each opened file. For multiple files, the plots are arranged in an `n x 2` (rows, columns) format where `n` is half the number of opened files.

1.2.2 Exporting Data

Through the File>>Export... submenu you can save the currently selected data item to file. The specific export type is selected directly as an option in the submenu. FIND currently has the ability to export to CSV, but any IO plugin may implement an export file type.

Note: This can be coupled with the Isolate Clusters tool (see Data Analysis section) to export clusters to external files in order to perform analysis and/or visualization with other programs.

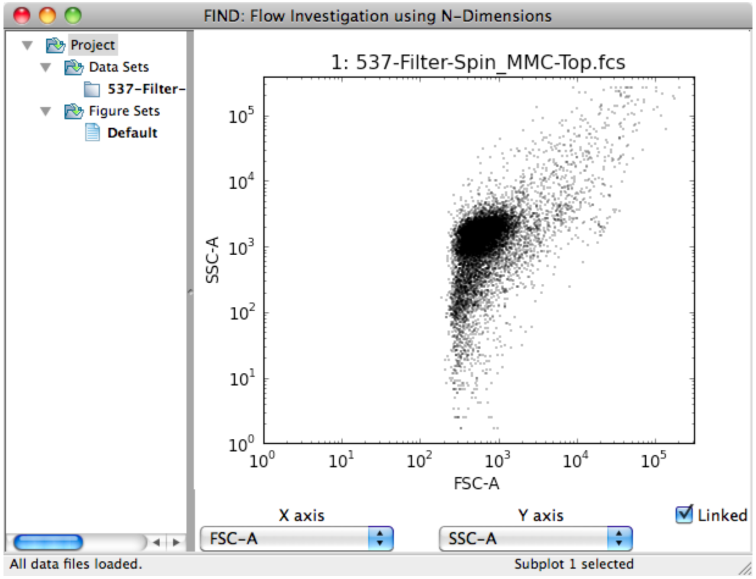
FIND: Flow Investigation using N–Dimensions
Project
Data Sets
537–Filter–
Figure Sets
Default
1: 537-Filter-Spin_MMC-Top.fcs
SSC-A
FSC-A
X axis
Y axis
Linked
FSC–A
SSC–A
All data files loaded.
Subplot 1 selected

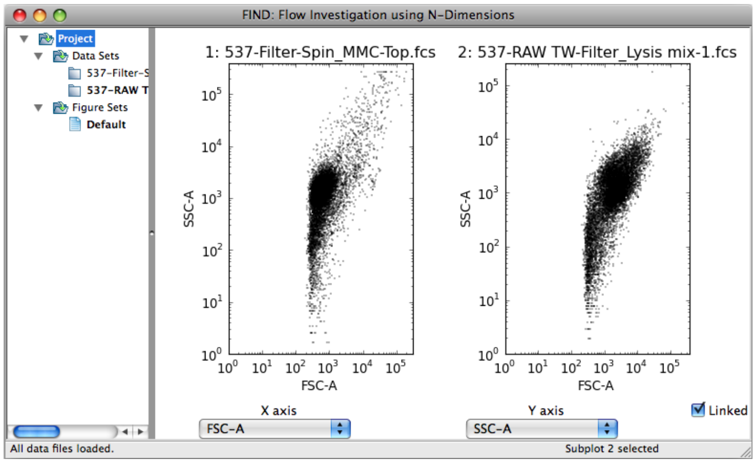
FIND: Flow Investigation using N–Dimensions
Project
Data Sets
537–Filter–S
537–RAW T
Figure Sets
Default
1: 537-Filter-Spin_MMC-Top.fcs
2: 537-RAW TW-Filter_Lysis mix-1.fcs
SSC-A
FSC-A
X axis
Y axis
Linked
FSC–A
SSC–A
All data files loaded.
Subplot 2 selected

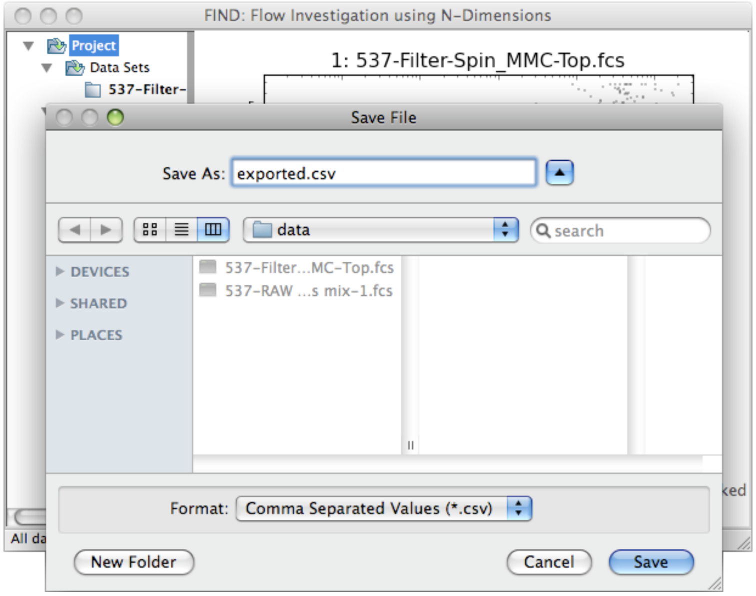
FIND: Flow Investigation using N–Dimensions
Project
Data Sets
537–Filter–
1: 537-Filter-Spin_MMC-Top.fcs
Save File
Save As: exported.csv
data
search
DEVICES
SHARED
PLACES
537–Filter...MC–Top.fcs
537–RAW ...s mix–1.fcs
Format: Comma Separated Values (*.csv)
New Folder
Cancel
Save

1.2.3 Saving and Restoring the Project

FIND allows you to save the entire state of the analysis as two files: one containing all the qualitative information about data structure, attributes, and visualizations, the second a simple binary file containing all the loaded numeric data. These two files can be packaged and transported in any manner for later use on the same machine or any other machine running FIND. To save your project, use the **File>>Save Project...** menu action (Ctrl-S on Windows or Command-S on OS X). The file you specify will be saved as a **.find** file and a **.npz** file. To load a previously saved project, use the **File>>Load Project** menu action (Ctrl-L on Windows or Command-L on OS X) and select the **.find** file of the project you want. Everything will be restored as it was when the project was saved.

1.3 Visualization

Data visualization in FIND is performed mainly through the plotting area, which makes up the right side of the main program window.

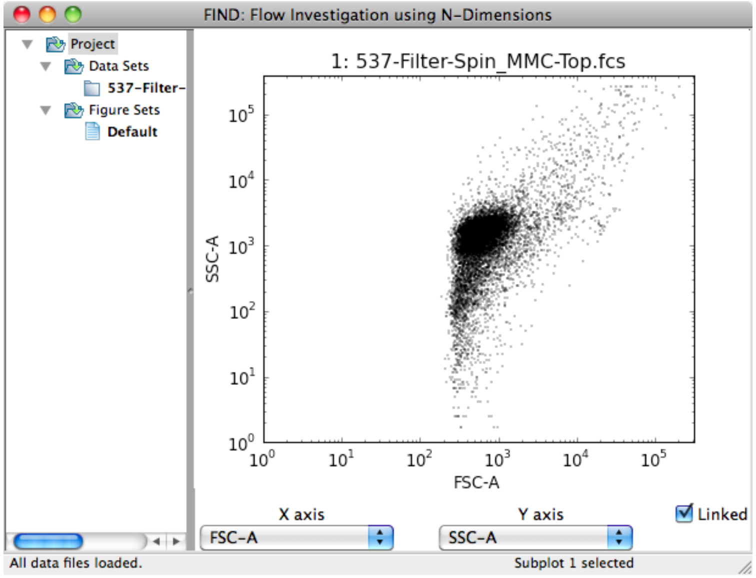

Here single data file has been opened, and a single 2D scatter plot automatically created, visualizing the first two dimensions of the data as given by the file. If multiple files had been opened, the plotting area would be automatically divided into `n x 2` (rows x columns), where `n` is half the number of opened files. The plots contained within the plotting area are collectively referred to as a Figure, which will be explained in detail later.

1.3.1 Plotting Data

First select the plot (also called subplot) you want by clicking on it. Your choice is reflected in the far right section of the status bar (see images below). A new plot can be created in the selected plot space by accessing the **Plot** context menu available by right-clicking (or ctrl-click on OS X for single-button mice).

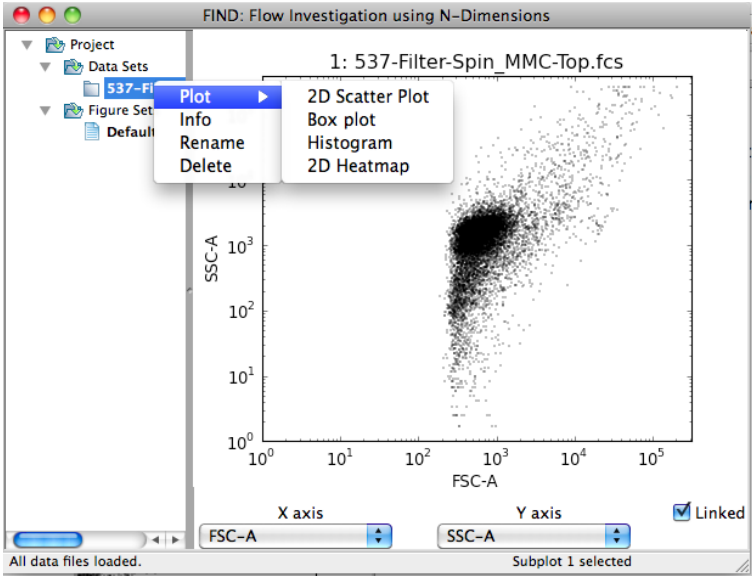
FIND: Flow Investigation using N-Dimensions
Project
Data Sets
Figure Set
1: 537-Filter-Spin_MMC-Top.fcs
Plot
Info
Rename
Delete
2D Scatter Plot
Box plot
Histogram
2D Heatmap
SSC-A
FSC-A
X axis
Y axis
Linked
FSC-A
SSC-A
All data files loaded.
Subplot 1 selected

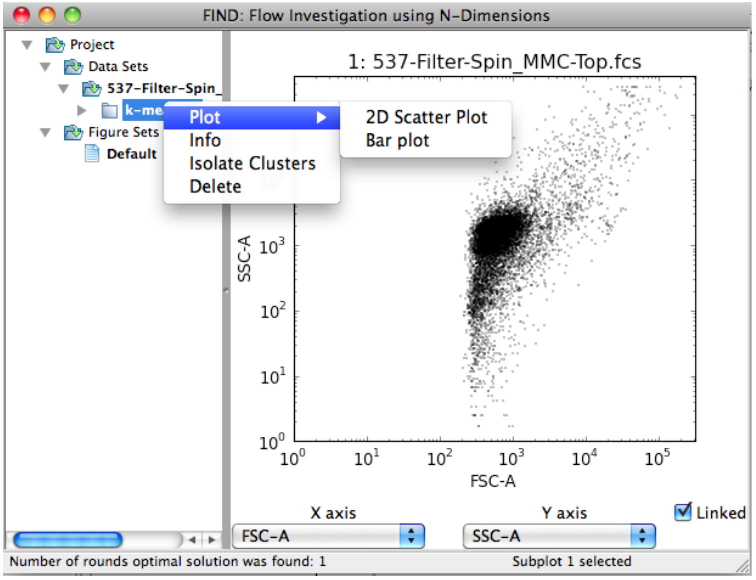
FIND: Flow Investigation using N-Dimensions
Project
Data Sets
Figure Sets
Default
1: 537-Filter-Spin_MMC-Top.fcs
Plot
Info
Isolate Clusters
Delete
2D Scatter Plot
Bar plot
SSC-A
FSC-A
X axis
Y axis
Linked
FSC-A
SSC-A
Number of rounds optimal solution was found: 1
Subplot 1 selected

The above images illustrate the subdivision of plot types. Specifically, plots are grouped into those applicable to datasets and those applicable to clusterings.

Note: Plot types applicable to both data items show up in the context menus for both (e.g. 2D Scatter Plot).

1.3.2 Adding and Deleting Plots

A new plot space can be added by selecting the **Plots>>Add Subplot** menu item. The new space is automatically selected as the current and so is ready to be drawn to.

A plot (and its space) can be deleted through the context menu (right-click) for that specific plot.

1.3.3 Altering Plots

The main method of interacting with and changing the view of a plot is through the Channel Selection panel beneath the plotting area. This panel contains two dropdown boxes labeled `X axis` and `Y axis` (when 3D plots are added a third for the Z axis will be added). Changing the selected channel (dimension) automatically updates all the plots in the plotting area, with some exceptions. Specifically, those plot types that display all dimensions at once, or are otherwise not designed to change will not be updated. Additionally, individual plots can be 'unlinked' from the selection panel. This involves first selecting a plot by clicking on it (within the area it is drawn to). The selection is confirmed by a change in the status bar at the bottom of the main window, indicating **Subplot n selected**. Next, deselecting the checkbox marked **Linked** causes the plot to be frozen as is with respect to the selection of dimensions. For example, a series of plots (Figure) could be created displaying all possible 2D plots of a dataset by creating one plot for each possibility, and progressively unlinking and changing dimensions for each.

The second method of changing the display of an individual plot is through changing the plot settings, accessible through the context menu when right-clicking on a plot.

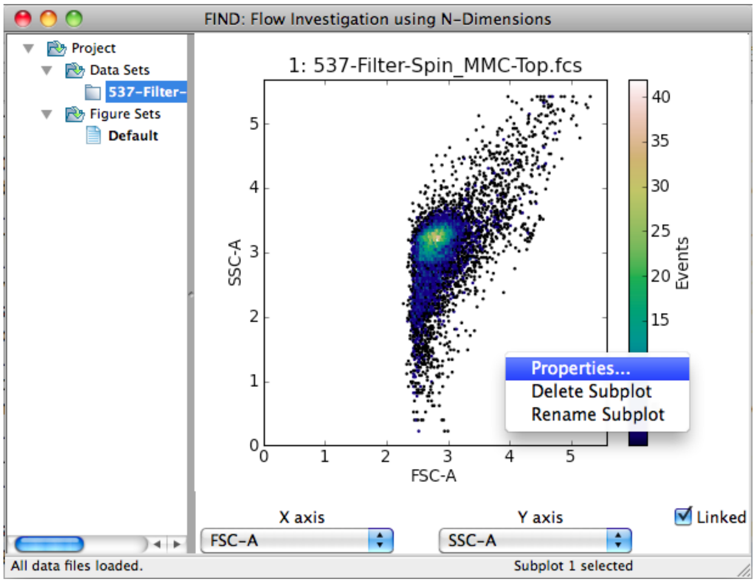

Selecting the **Properties...** menu item displays a settings dialog allowing you to alter the display or calculation with options specific to the plot type or general. Examples of each and more information are discussed in the following sections.

General Plot Properties

FIND provides two sets of general plot options that appear where appropriate (and available to plugin authors). First, below, is the Plot Range options panel. Here you can modify the graph window within which the data is displayed. By default, FIND plots on both axes, a range from 0 to the maximum value of the data plus five percent, i.e. `(0, max(data) * 1.05]`. This is indicated by the selection of the **Auto** checkboxes.

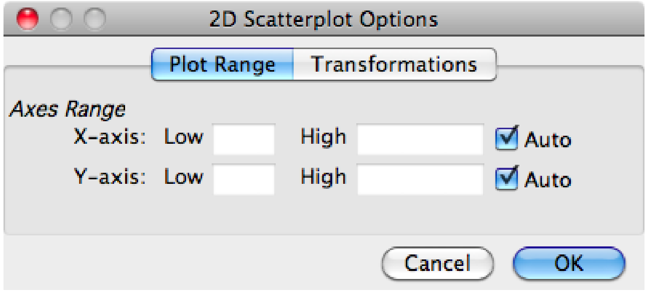

The second set of general options is the Transformations panel. This setting is initially set automatically by inspection of the data. FCS format files list the amplification type (linear or logarithmic) used to capture each channel of data, and FIND uses this information to decide whether the data should be displayed in a linear or $\log_{10}$ scale. In this options panel, you can tell FIND to either use automatically discovered information, or choose what scale the data are displayed in for each axis independently.

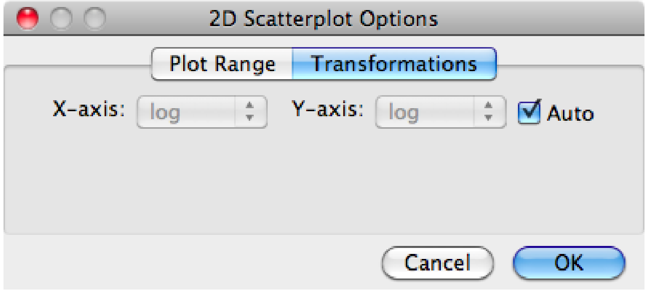

1.3.4 Dataset Plots

FIND currently provides four plot types for visualizing datasets: 2D Scatter Plot, Box Plot, Histogram, and 2D Heatmap. Each of these plots are explained in the following sections.

2D Scatter Plot

The 2D Scatter Plot draws a single point in two dimensional space for each event (cell) in the dataset. Below is an example plot of the Forward Scatter (x-axis) and Side Scatter (y-axis) on a $\log_{10}$ scale. As seen in the

General Plot Properties section above, there are no options particular to the 2D Scatter Plot.

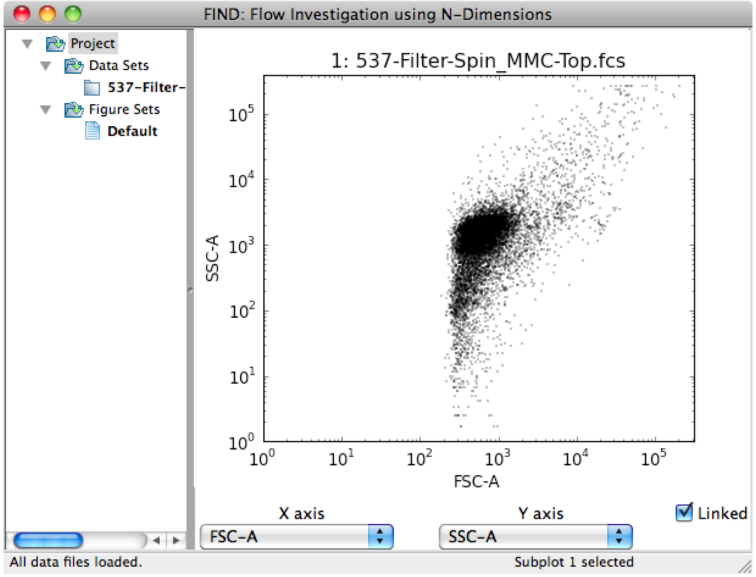

Boxplot

The Boxplot visualization is an example of a plot that is insensitive to user changes to the displayed channel, as it displays data for all channels at once. The x-axis here displays one tick-mark for each channel in the data, and for each channel a traditional box plot in the y-axis.

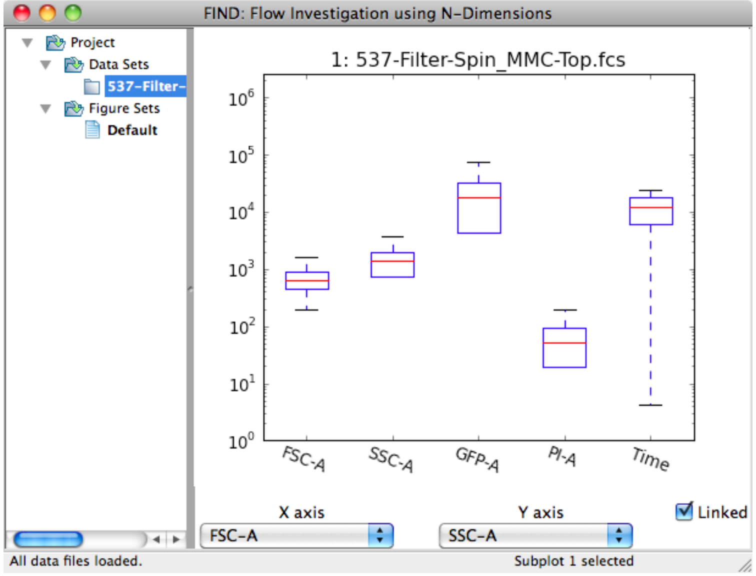

The only alterable properties for this plot (as seen below) is the angle to which the x-axis labels are rotated, with `0` representing a horizontal orientation. This is useful for datasets with many channels where FIND does not adequately choose an angle that cleanly separates the labels.

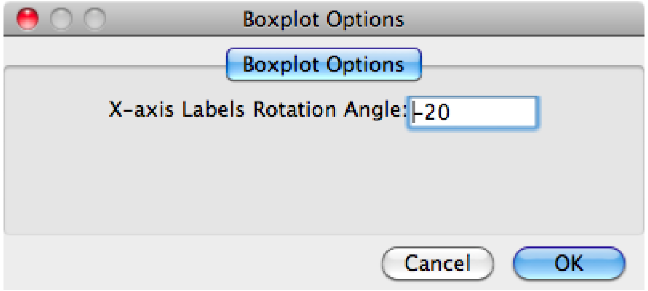

Histogram

The Histogram plot displays a single channel (x-axis) and, by default fits a Gaussian kernel to the data as an approximation to get the smooth curve seen in the image below.

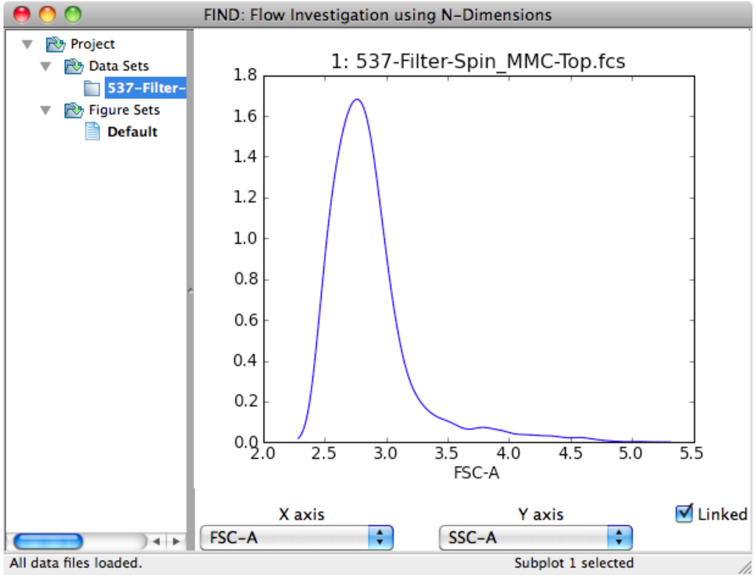

The Gaussian kernel estimation gives a good representation of the overall shape of the data, but may not adequately estimate the amplitude. In the options for this plot, you can additionally select to display the histogram as a traditional binned plot separately, or overlay the estimation with the binned version via the **Histogram Type** option. Finally, you can set the fineness of the plot by changing the **Histogram Bins** option.

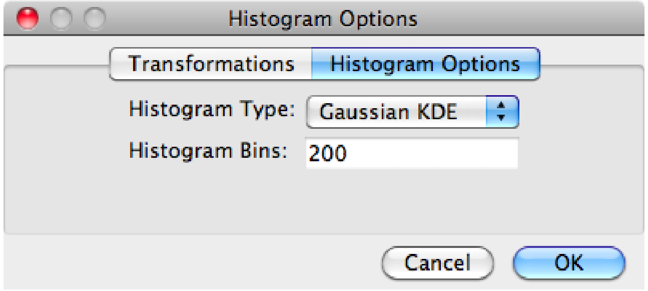

2D Heatmap

The heatmap plot is essentially a 2D histogram. It divides the plot into a set of hexagonal bins, only displaying a bin if it contains at least one data point. The density of points contained within the bin is displayed as a color map (heat map) with a scale bar on the right side of the plot.

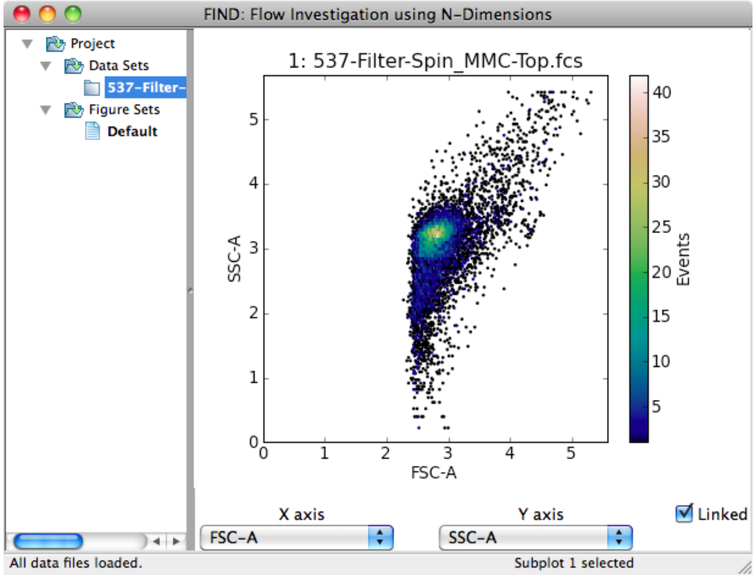

There are three (currently two) modifiable options for the 2D Heatmap. The **Heatmap Type** is currently under development and other options will eventually be available. The **Color Map** option sets the range of colors that are mapped to bin density from low to high. The default gist_earth (seen in the above image) is generally good, but other color maps may provide better visualization for sparse or especially dense datasets. The **Bins** option specifies the fineness of the 2D subdivision of the data points. Larger values may provide better insight into the data, but will take longer to plot.

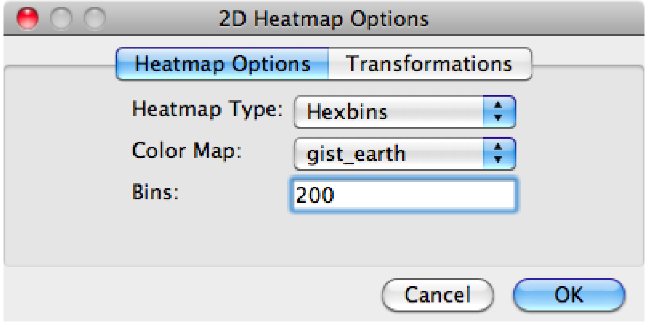

1.3.5 Clustering Plots

FIND currently provides two plot types for visualizing clustering results: 2D Scatter Plot and Barplot.

2D Scatter Plot

The only difference between the the 2D Scatter Plot as applied to a clustering as opposed to a dataset, is color. Each data point is colored according to cluster membership as seen in the image below. There are no plot-specific options.

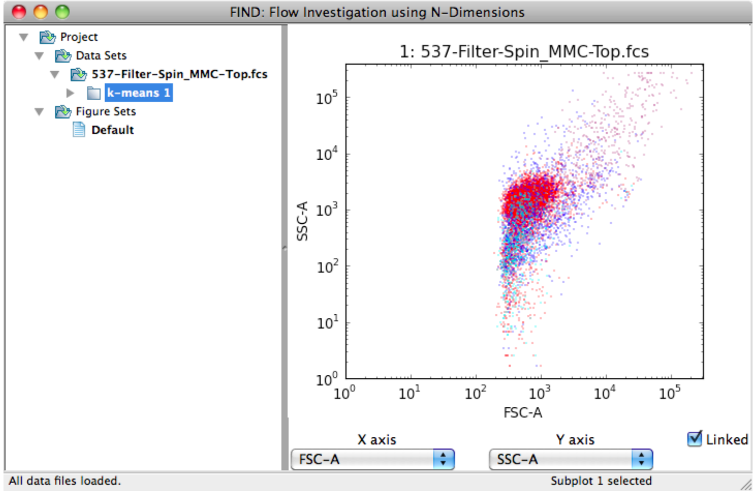

Barplot

This plot displays cluster membership counts in a vertical bar along the y-axis. Each cluster is a tick on the x-axis. The default y-axis value is the percentage of each cluster out of the total events in the parent dataset.

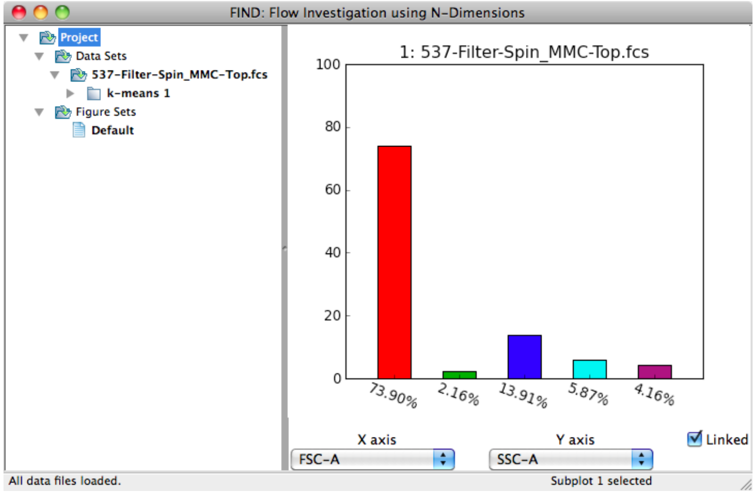

The Barplot comes with two plot-specific options. The first option allows you to change the rotation angle of the x-axis labels (similar to the Barplot). The **View** option changes the meaning of the y-axis values: the default percentage (as mentioned above), the percentage of the top level parent, and the total number of events in each cluster (no percentage).

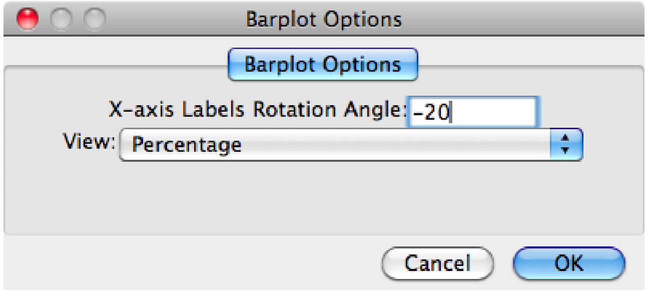

Note: As will be explained in the section of the documentation on clustering, new dataset items can be created by isolating multiple or individual clusters. These new datasets appear as children of the original **parent** dataset. As these are dataset items just like those created by opening files, they can be clustered as well. So choosing the **top level parent** option for the **View** will calculate the percentage by making the denominator the number of events in the original parent dataset instead of the dataset the clustering was created from.

1.3.6 Figures

A Figure collects everything within and related to the plotting area. Specifically: all plots (and their settings) within the plotting area, the layout of the plots, the selected channels, and the linked/unlinked status of each plot.

The list of created Figures is in the Project tree (on the left side of the FIND window) under the Figure Sets group. At startup, FIND creates a single *Default* Figure that all plots will initially be created in.

If you want to rename or delete a Figure, access the context menu for it.

Note: There must be at least one Figure, and FIND will not allow you to delete the current (in bold) Figure.

Plotting Area Setup

The plotting area is organized into a rectangular grid. Initially, the grid layout and the number of plots is determined by the number of opened files, as discussed earlier. If you want to change the number of plots or the number of rows and columns, you must use the **Plots>>Setup** menu option.

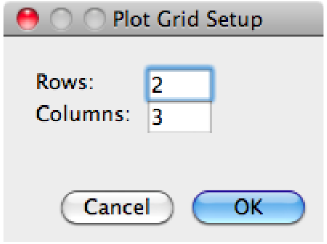

Below is an example of a `2 x 3` grid setup:

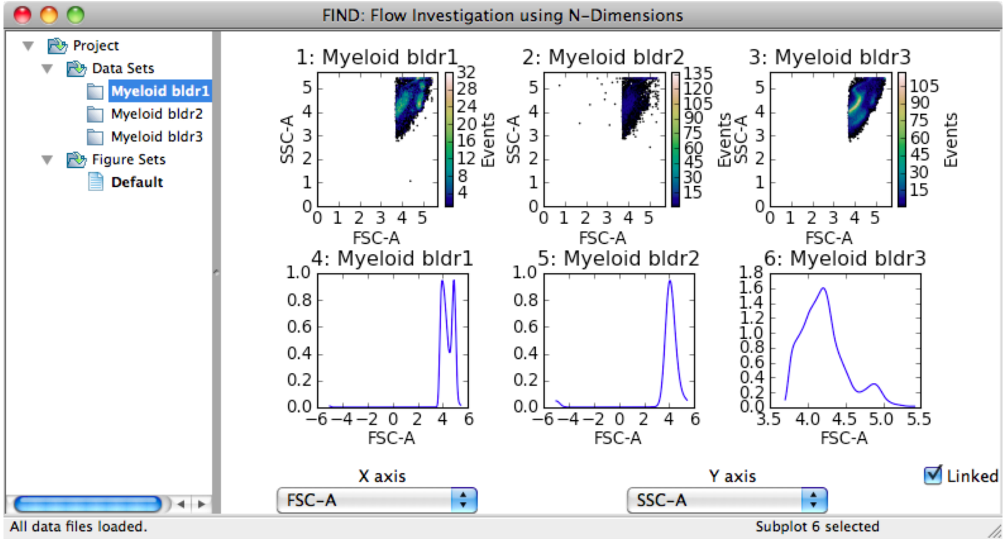

Creating Figures

A new Figure can be added through the **Plots>>Add New Figure** menu item. This will first ask you to type a name for the new Figure. Then the new Figure will appear in the Project tree. It is automatically selected (shown in bold), a single subplot is created and the currently selected data item is plotted by default with a 2D scatter plot (if it has a clustering, that will be plotted).

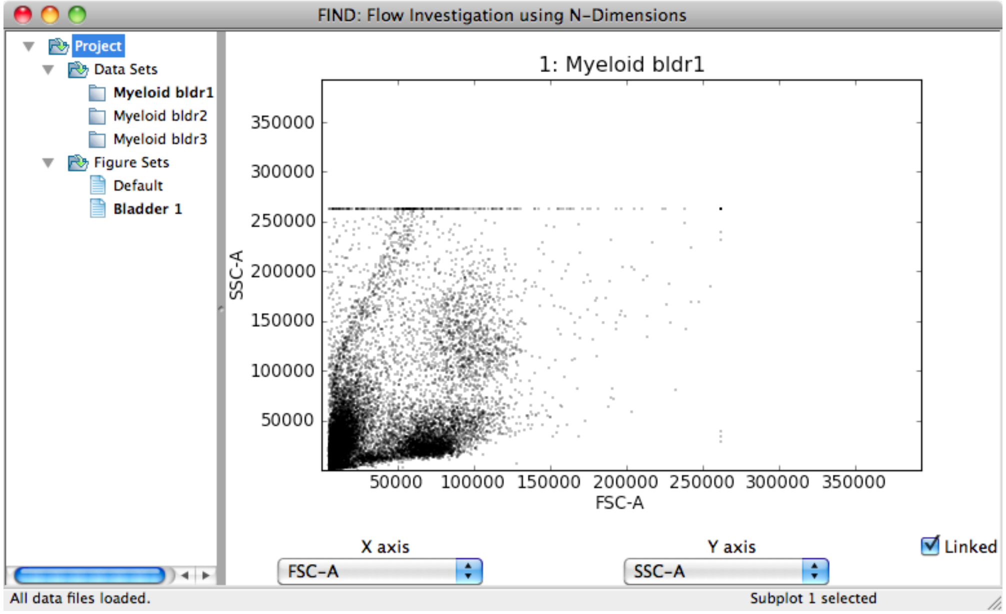

Switching Figures

If you have created multiple Figures, switching between them is as simple as clicking on the Figure item in the Figure Sets group. This action will completely replace the contents of the plotting area with the contents of the new Figure.

Note: For larger datasets (100K events or more) switching between Figures may take a few seconds since FIND has to recalculate and draw all plots saved into the Figure.

Exporting Figures

FIND enables you to save the contents of the plotting area to a number of standard image formats: PNG, PDF, PS (post-script), EPS, and SVG. To perform an export, use the **Plots>>Export Figure...** menu item. A save dialog will appear and ask you to specify the name of the file and the file type of the new image file. An example of a Figure export is below:

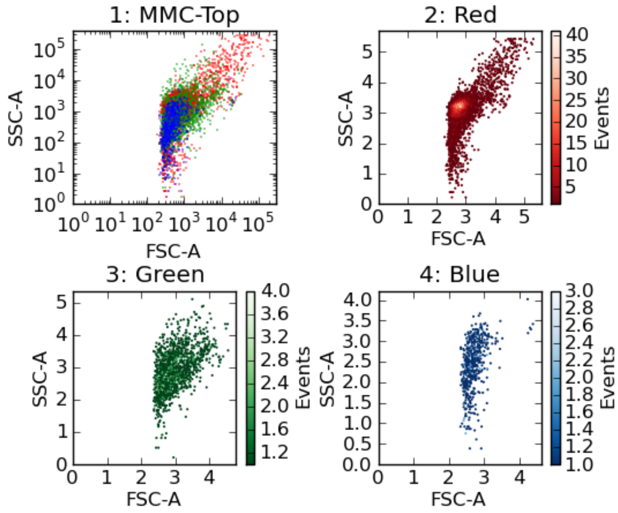

1.4 Data Analysis

Moving from analysis using traditional gating techniques to automated and semi- automated methods such as are provided in FIND can be a difficult shift due to the degree of control you give up over how the data is divided into separate clusters or segments. This section will provide an overview of the methods FIND provides for analysis, how to use them, and suggestions on how to proceed given those tools.

1.4.1 Clustering

Clustering or machine learning describes the process of mathematical analysis of the given data in order to determine groupings of data points (events) that are more similar to each other than to those not in the group. These algorithms are split into two categories based on how they work:

1. Supervised - Generally require some sort of "training" data where the classification (group) of each point is known ahead of time so the algorithm can use that data to "learn" and be able to predict future datasets.

2. Unsupervised - Requires no foreknowledge of any kind. Simply attempts to classify data based on mathematical relationships (such as distance) between the input data points.

Clustering with FIND

FIND currently provides two unsupervised clustering methods: k-means and a modified k-means specifically designed for Flow Cytometry data as published by T. Bakker Schut et al. in *Cytometry Part A*.

All built-in clustering algorithms are available through the **Cluster** menu. Plugin clustering algorithms are available in the **Plugins>>Cluster** menu. When you choose an algorithm from the menu, it operates only on the currently selected dataset (bold in the Data Sets subtree; see Introduction section on the User Interface). So make sure the dataset you want to analyze is selected before you run a clustering. When the clustering is completed, a new entry is added as a child of the dataset in the Project tree. The default name is the name of the clustering method and an auto-incremented integer (e.g. k-means 1). Currently you cannot change the name of a clustering entry. Each clustering appears with an expand arrow next to it. Clicking on the arrow will show a text entry underneath the clustering with a list of the options and the values you provided to run that particular clustering instance. This can be useful if you cluster run multiple clusterings, either on the same dataset or different ones, to make sure you used the same settings.

Note: Many clustering methods use random number generation to perform certain aspects of the algorithm such as choosing the initial cluster centers in k-means. This tends not to greatly affect the final results and, for example, in k-means, the user can specify that the algorithm be run multiple times to reduce any potential effect. Ultimately, just be aware that you may not get the *exact* same results running the same algorithm twice, but there should be no significant difference between runs.

The basic requirement for many unsupervised clustering methods is that you provide the number of desired clusters or groups you want the data to be partitioned into. If your data is fairly well separated/distinguished between different cell types, you may want to enter the number of different types you expect. Often, however, it can be helpful to request a larger number of clusters than cell types that you expect. This can give you a finer understanding of the layout of the data, to which you can apply your domain knowledge to throw away, keep, or combine into larger groups (this tool will be described later) the initially found clusters.

K-Means Clustering

For a description of the operation of the basic k-means algorithm, please see the excellent Wikipedia article. Note that while this algorithm was not designed specifically for Flow Cytometry data, it has been successfully applied to such data (citation).

Below is the options dialog that appears when you select the **Cluster>>k-means** menu item.

- **Number of clusters** - The number of clusters that should be found as the final result of the algorithm
- **Center calculation** - The mathematical measure the algorithm should use when determining the cluster center from its members.
- **Number of passes** - The algorithm is re-run a number of times specified by this parameter, each time a new set of initial cluster centers are chosen. The results of each run are compared to find the best.

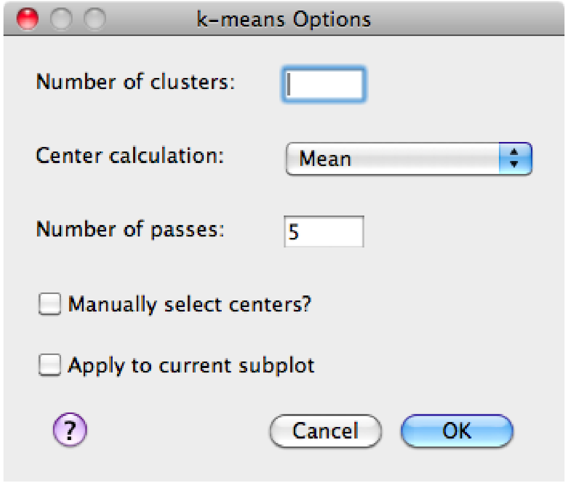

- **Manually select centers** - This option allows the user to (in a separate window that pops up) to choose the initial centers of the clusters by clicking within the data space in any set of 2 dimensions. Choosing this parameter will remove the need to specify the number of target clusters as well as the option to re-run multiple times. Consequently, those options are disabled when this option is checked.
- **Apply to current subplot** - Upon completion of the clustering, the result will be displayed as a colored scatterplot, replacing the currently selected plot.

Bakker Schut K-Means Clustering

As mentioned earlier, this algorithm was implemented from the description provided in the publication by T. Bakker Schut et al. in *Cytometry Part A*.

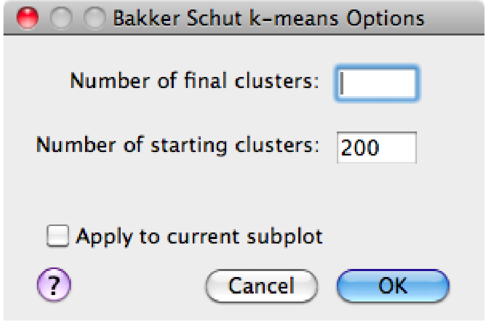

- **Number of final clusters** - The number of clusters that should be found as the final result of the algorithm.
- **Number of starting clusters** - The algorithm begins by selecting a large number of clusters (200 by default) and iteratively merging those closest together until the specified target number of clusters is acheived. This number affects performance, so if the clustering is running too slowly, decreasing this parameter will help.
- **Apply to current subplot** - Upon completion of the clustering, the result will be displayed as a colored scatterplot, replacing the currently selected plot.

1.4.2 Interacting with Clusterings

After running a clustering algorithm on a dataset, FIND provides a number of means to analyze and interact with the clustering. The most basic is visualization of the clustering; these options are discussed in the previous section of this manual. Other options are provided through the **Data** menu and the context menu available by right-clicking on the clustering item in the Project tree; these options are discussed below.

Clustering Information

The first option available, after plots, in the context menu for clusterings is the **Info** item. Selecting this option provides a dialog indicating, for each cluster: the color, the percentage of events belonging to that cluster out of the whole for the parent dataset, and the total number of events the percentage translates into.

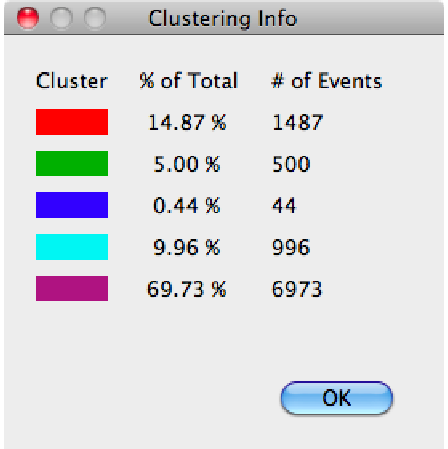

Isolating Clusters

The next available option through the context menu is **Isolate Clusters**. This incredibly useful tool allows you to create an entirely new dataset by choosing one or more clusters from the selected clustering. This is accomplished through the dialog show below:

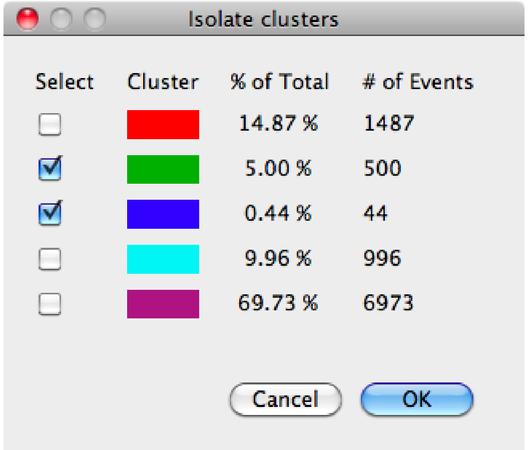

This dialog is essentially identical to the clustering information dialog, with the added column of check-boxes. Selecting one or more checkboxes will create a new dataset with the selected clusters joined together. Once you select the cluster(s) you want, clicking the OK button will bring up a simple dialog box asking you to give a name to the new dataset. When you type in a name and click OK again, the new dataset will appear as a child in the tree of the dataset it was created from (parent dataset).

Note: As mentioned in the Data section, datasets which represent isolated clusters can be exported as external files to disk, allowing visualization and analysis of clusters in other programs.

Recoloring Clusterings

As discussed earlier, there is some randomness in certain clustering algorithms. For example, the initial cluster centers may be chosen randomly. These centers move around as the algorithm progresses, but their order determines the color the cluster is represented by. Thus, even if two runs of the same algorithm produce the same clusters for a dataset, the order they are presented in may be different. The results are identical, but they are presented to the user in different colors, making it more difficult to do comparative analysis. The **Data>>Recolor Clusters** menu action provides a means to fix this problem.

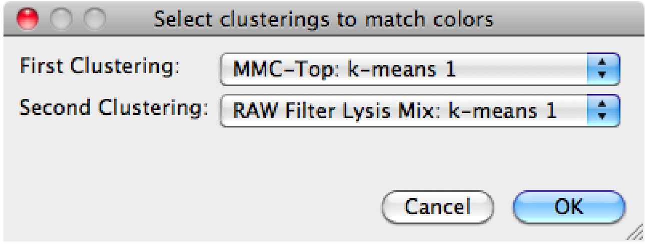

In the dialog shown above, select the two clusterings you are interested in. FIND then compares each of the clusters between the two selected items to determine the cluster pairs that are most similar. The clusters are then reordered such that those most similar have the same color. The following two images illustrate this exact point. Two clusterings of the same data produce three clusters with the exact same percentage breakdown, but the blue and red clusters appear to be switched. After recoloring, in the second image, the clusters are ordered correctly as can be seen in both sets of graphs.

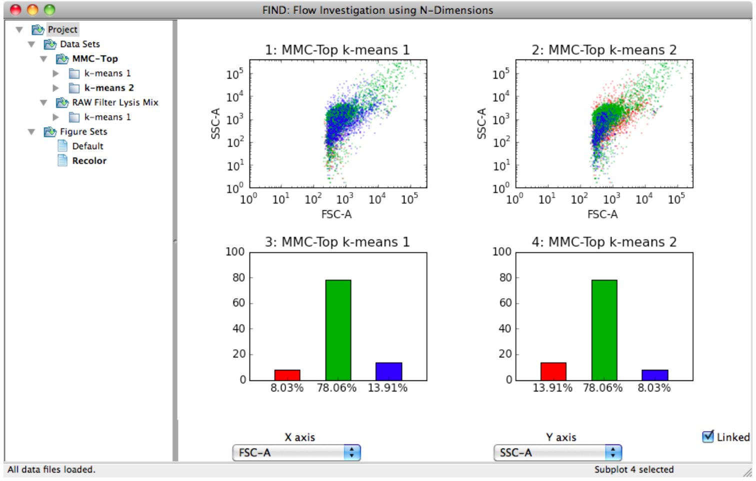

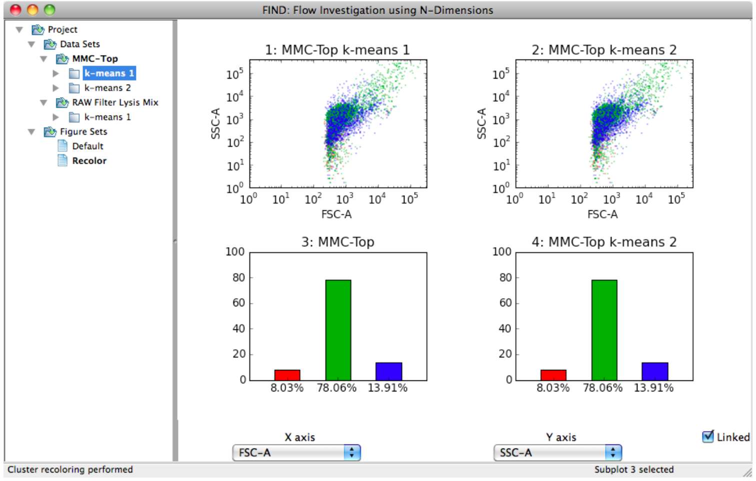
FIND: Flow Investigation using N-Dimensions
Project
Data Sets
MMC-Top
k-means 1
k-means 2
RAW Filter Lysis Mix
k-means 1
Figure Sets
Default
Recolor
1: MMC-Top k-means 1
2: MMC-Top k-means 2
SSC-A
FSC-A
3: MMC-Top
4: MMC-Top k-means 2
8.03%
78.06%
13.91%
X axis
Y axis
Linked
Cluster recoloring performed
Subplot 3 selected

CHAPTER

TWO

FIND DEVELOPER DOCUMENTATION

Contents:

2.1 Introduction

Developers are encouraged to create additional functionality for FIND through the available plugin architecture. Specifically, FIND allows for plugins in the following categories: Analysis, Clustering, Graphing, IO, and Transforms. Each of these plugin types are described in detail with code examples in their respective sections later in this documentation.

Beyond the API provided within FIND (which will be discussed where appropriate per plugin type), the following external Python libraries are available to developers:

- Numpy (http://numpy.scipy.org)
- Scipy (http://scipy.org)
- Matplotlib (http://matplotlib.sourceforge.net/)
- wxPython (http://wxpython.org/)
- Pycluster (http://bonsai.hgc.jp/~mdehoon/software/cluster/software.htm)

2.1.1 General Plugin Architecture

In order for a plugin to be recognized, the main module file containing it must be placed within the appropriate folder in the Plugins directory that must accompany the FIND executable. For example, included with the FIND distribution is a clustering plugin called **Unicluster**. The plugin consists of one file `unicluster.py` which is placed within the `cluster` folder. Internally, module files must provide two items, indicating to FIND the existence of plugin functionality. The first is a registration method which in all code examples follow the naming convention `method_register()`. This method returns a `tuple` that provides FIND with references to necessary methods or information specific to the plugin type. Given that the register method can take any name, modules must indicate its existence to find by placing it in the `__all__` magic list variable provided by Python. The FIND plugin architecture will use this list and the corresponding methods to internally link to the provided plugin functionality. Plugins may consist of multiple modules, but only modules with the `__all__` variable defined will be examined for plugin functionality. The specifics of what each registration method must provide are discussed in the appropriate

section of the manual. Finally, analysis, graph, and transform plugins must provide string identifiers such that authors of other plugins can easily incorporate the functionality of other plugins within their own code. For example, a useful analysis plugin may provide functionality that the author of a graphing plugin has an interesting way of displaying through a plot. The specifics of how to call these plugins can be found within the appropriate subsection of this manual.

2.2 Analysis Plugins

This category of plugins attempts to provide a more generic means of including additional functionality within FIND. Currently, analysis plugins can fill two roles: Provide some visual analysis results when a user clicks on the analysis plugin from within the plugins menu, or return numeric analysis results to a piece of code calling it from within FIND. Analysis plugins must at least provide numeric results to callers, but the plugin can also indicate that it has the ability to display some results to the user through a dialog or other window object. This is possible through the method registration function which should be similar to the following:

```
def analysisMethod_register():
   return (analysisMethod, True)
```

Here, the first item in the tuple is a reference to the provided analysis method, while the second item is a boolean indicating (True in this case) that this method is callable by the user and will display some results visually. A True in this slot will cause FIND to create an enabled menu item in the Plugins>>Analysis menu. A False will cause FIND to place a disabled menu item to simply inform the user the plugin was loaded.

The analysis method signature and doc string are as follows:

```
def analysisMethod(data, **kwargs):
   """
   string-ID; method-name; Method description string
   """
   ...
   ...
   return (result, message)
```

The `data` parameter is an `m x n` array (numpy `ndarray`) with `m` data points (events), and `n` dimensions (channels). The `**kwargs` parameter is a dictionary containing options for the analysis algorithm. These could be either generic options provided by FIND or specific options you specify and publish for others to use. Currently FIND provides one option in the args dictionary: `'parentWindow'`. This provides a reference to the FIND main window, and allows the analysis method to show a dialog or other window class for the purpose of displaying results to the user.

The first line of the doc string must be semicolon-separated into three fields as seen above. The 'string-ID' field is what other plugin authors will give to the analysis module in order to access the analysis method from within their own code. The 'method-name' is a short name that FIND will use for the menu item placed in Plugins>>Analysis. The final field will appear in the program status bar when a user moves the mouse over the menu item for the analysis plugin.

The return of the analysis method consists of a 2-tuple. The first element of the tuple is some result (or None) if it is being called from code and not by user interaction with the Plugin menu. The second element is a

string message that will be displayed to the user in the program status bar once the method has completed running.

Plugin authors will be able to make use of analysis plugin methods by passing the string identifier in the following manner to the Analysis module:

```
import analysis.methods as am
result = am.getMethod('pca')(data)
```

2.3 Clustering Plugins

Clustering algorithms are certainly a type of analysis, but their importance in relation to analysis of Flow Cytometry data is such that they have been given their own category.

The functionality of clustering plugins is divided into two parts:

- Options dialog
- Clustering algorithm

Both parts are required, and must be returned by the registration function that must be included in the `__all__` variable. For clustering plugins, FIND expects the registration method to return a 2-tuple containing the clustering function and the options dialog class. For example, the `unicluster_register` method of the Unicluster plugin is:

```
def unicluster_register():
    return (unicluster, UniclusterOptionsDialog)
```

Note: Only the method/class *reference* is passed, so () are omitted.

2.3.1 Options Dialog

The options dialog provides a graphical interface for users to modify the behavior of the clustering algorithm by providing a set of input options.

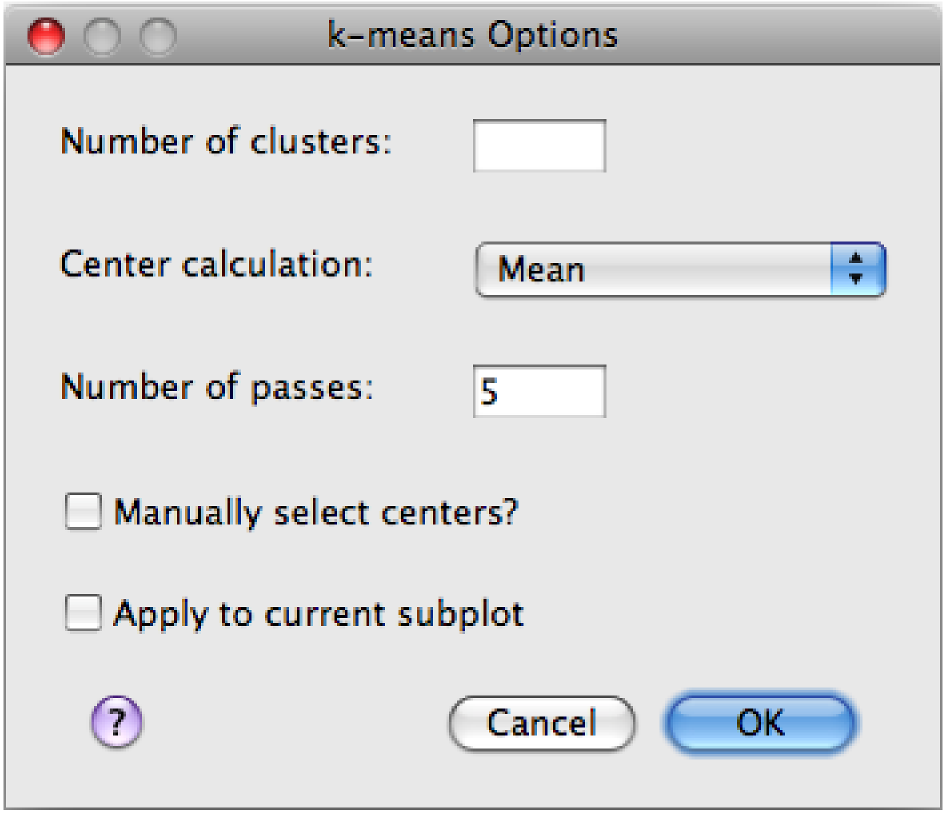

In code, the dialog is nominally a subclass of wx.Dialog, which provides the basic windowing functionality. However, FIND provides a template class (`ClusterOptionsDialog`) in the `pluginbase.py` module in the root plugins directory which is highly recommended for use as a superclass of the plugin options dialog. The `ClusterOptionsDialog` class already subclasses `wx.Dialog` and provides a set of methods that FIND expects when interrogating the dialog class for information. Specifically these are `getMethodArgs()` and `getStrMethodArgs`. The first returns a dictionary of option/value pairs that will be passed through the `**kwargs` parameter to the clustering algorithm. The second provides an equivalency between the internal options variable names and string descriptions of the options that can be presented to the user. Further details and code are available in `pluginbase.py` and `unicluster.py` in the cluster folder of the plugins directory.

2.3.2 Clustering algorithm

This method provides the multidimensional analysis for grouping the input data into class groupings. The function specified in the register method is called by FIND after the user selects the clustering plugin and provides input to the options dialog. The expected method signature, doc string, and return are:

```
def clusteringMethod(data, **kwargs):
    """
    ClusteringMethod name; Description of ClusteringMethod

    Any additional comments
    """
    ...
    ...
```

```
    return (cluster_IDs, message)
```

The `data` parameter is an `m x n` array (numpy `ndarray`) with `m` data points (events), and `n` dimensions (channels). The `**kwargs` parameter is a dictionary containing the algorithm options and values specified by the user in the options dialog. It is in this manner that the options dialog is connected to the algorithm function, allowing the user to alter the algorithm behavior.

The function doc string must contain on its first line, a specially crafted string containing the display name for the clustering method and a description of the method, separated by a semicolon. When importing the plugin, FIND will parse this first line for these two items, and incorporate them into the interface. Specifically, the method name will be used for the menu item in the Plugins menu, and the description will be displayed in the program status bar when the user places the mouse over the method item in the menu.

At the end, FIND expects a 2-tuple return containing an `m x 1` array and a `str`. The array contains integers `0...k` (where `k` is the number of assigned clusters), such that each entry corresponds to the cluster assignment for that data point in the original input data array. The second part of the tuple is a string message that will be displayed to the user in the program status bar upon completion of the algorithm.

2.4 Graphing Plugins

Graphing plugins provide the main visual data exploration method for the user. All graphs/plots are fundamentally provided by the external library matplotlib so developers should familiarize themselves with the functionality it provides.

The functionality of graphing plugins is divided into two parts, similar to the clustering plugins:

- Options dialog
- Graphing method

Both parts are required, and must be returned by the registration function that must be included in the `__all__` variable. For graphing plugins, FIND expects the registration method to return a 3-tuple containing the graphing method, the options dialog class, and a list containing the data item types the plugin applies to. Currently there are two data items: datasets and clusterings, represented by `0` and `1` respectively. For example, the `histogram_register` method of the Histogram plugin is:

```
def histogram_register():
    return (histogram, HistogramOptionsDialog, [0])
```

Note: Only the method/class *reference* is passed, so () are omitted.

Here the third item of the tuple is a list containing one entry: `0`. This indicates that Histogram plots are only applicable to datasets and not clusterings. For a graphing method that applies to both (like the built-in 2D Scatterplot) the list would be `[0,1]`.

2.4.1 Options Dialog

The options dialog provides a graphical interface for users to alter the output of the graphing plugin.

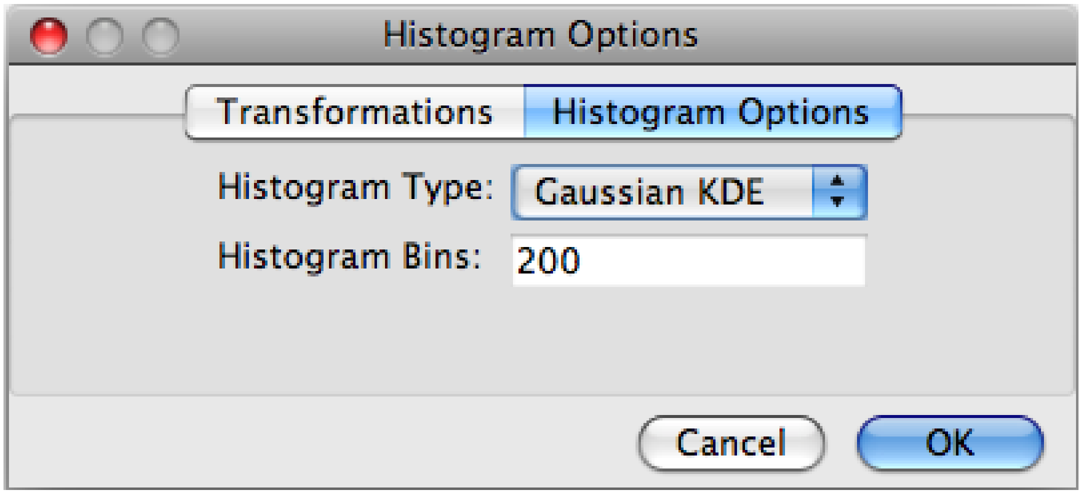

The FIND API provides a useful base class for creating options dialogs for graphing methods: OptionsDialog. This class subclasses wx.Dialog, and provides a wx.Notebook control that gives the tabs seen in the figure above. The class is available by the following import statement:

```
from plot.base import OptionsDialog
```

The base OptionsDialog class provides, in addition to the tabs, a built-in validation routine and template, as well as a template for loading and providing the options associated with the overall dialog:

```
class OptionsDialog(wx.Dialog):
   def __init__(self, parent, subplot, title="Plot Options", size=(200,180)):
      wx.Dialog.__init__(self, parent, wx.ID_ANY, title, size=size)
      self.CenterOnParent()

      self.NB = wx.Notebook(self)

      # sizer for layout
      sizer = wx.BoxSizer(wx.VERTICAL)
      sizer.Add(self.NB, 1, wx.EXPAND)
      self.SetSizer(sizer)


   def cmdOK_Click(self,event):
      """
      Generic handler method for capturing the OK button click. This method
      will run the validate method for each panel in the Notebook and collect
      any error messages. If errors exist, a message dialog will be shown
      and the options dialog will be prevented from confirming until the
      errors are fixed. Otherwise, the event will be bubbled up as normal.
      """
      msg = []
      for i in range(self.NB.PageCount):
         msg.extend(self.NB.GetPage(i).validate())

      # Display any error messages
      if len(msg) > 0:
          dlg = wx.MessageDialog(None, '\n'.join(msg),
                                 'Invalid input', wx.OK | wx.ICON_ERROR)
```

```
            dlg.ShowModal()
            dlg.Destroy()
            return

        event.Skip()

    def loadOptions(self, opts):
        """
        Loads saved options into all notebook pages via the loadOptions()
        method that must exist in each.

        Unless specific functionality is needed, subclasses of OptionsDialog
        should not need to override this method.

        :@type opts: dict
        :@param opts: The saved plot options.
        """
        for i in range(self.NB.PageCount):
            self.NB.GetPage(i).loadOptions(opts)

    @property
    def Options(self):
        """
        The Options property will retrieve all settings from each notebook page in self.NB

        Unless specific functionality is needed, subclasses of OptionsDialog
        should not need to override this property.

        :@rtype: dict
        :@return: The specified plot options.
        """
        options = {}
        for i in range(self.NB.PageCount):
            options.update(self.NB.GetPage(i).Options)

        return options
```

In this class, the `loadOptions` method and the `Options` property are run automatically and do not need to be overridden by subclasses. The `cmdOK_Click` method is available for automatic validation, if desired. However, if you want to include custom code in the click event method for the OK button on the Dialog, you must implement your own. However, you can still make use of the validation code by calling the `cmdOK_Click` from the superclass once your custom event code is completed.

Separate groupings of options are placed in tabs, and some general sets of options are provided by the FIND API.

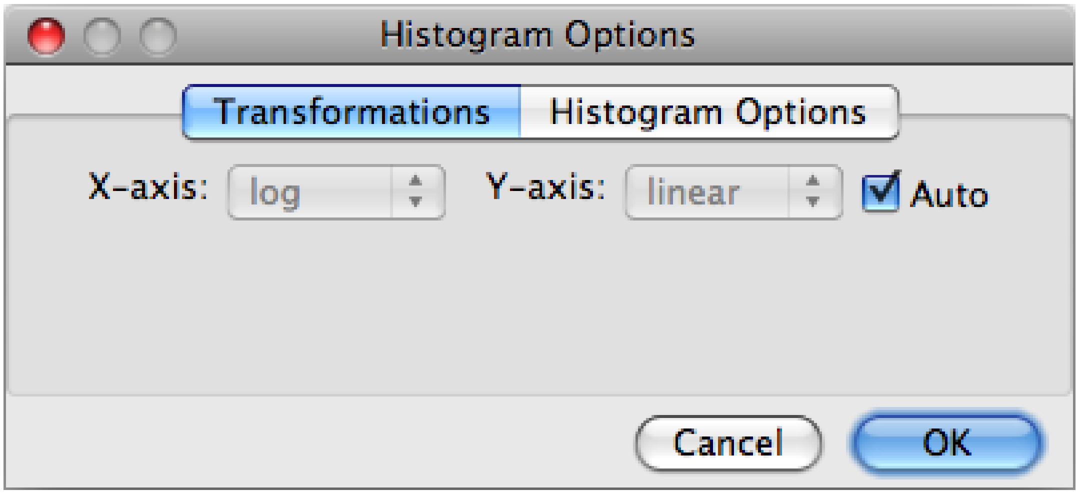

For example, the Transformations options (as seen in the figure above) can be accessed by the following import statement:

```
from plot.base import TransformOptionsPanel
```

Developers can provide their own option panels that can be included as a tab in the dialog by subclassing from the `OptionsPanel` class, also available in the `plot.base` module.:

```
class OptionsDialogPanel(wx.Panel):
   def loadOptions(self, opts):
      pass

   def validate(self):
      pass

   @property
   def Options(self):
      pass
```

Each of these methods must be overridden for each panel that subclasses from `OptionsDialogPanel`. The `loadOptions` method simply takes the `opts` dictionary and copies the panel-specific options from it into the panel controls. The `validate` method builds a list of strings containing error messages, one list item per validation error. These lists are gathered from each options panel, concatenated, and displayed to the user after clicking the OK button, and control is returned to the user for them to correct the errors. If none of the panels report errors, program execution continues to the graphing method. The `Options` property is the opposite of the `loadOptions` method; it builds a dictionary of option/value pairs from the controls on the panel. Each panel contributes its own options dictionary to a larger one that is finally passed to the graphing method for use.

The other built-in, generic options panels are:

```
RangeOptionsPanel, SingleTransformOptionsPanel
```

The RangeOptionsPanel allows users to specify range in the X and Y axes. The SingleTransformOptionsPanel is identical to the TransformOptionsPanel, except it provides a single transformation for the whole plot (instead of by axis). Below is a list of the options each of the previously mentioned options panels provides:

```
RangeOptionsPanel: 'xRange', 'yRange', 'xRangeAuto', 'yRangeAuto'
TransformOptionsPanel: 'xTransform', 'yTransform', 'transformAuto'
SingleTransformOptionsPanel: 'transform', 'transformAuto'
```

For the `RangeOptionsPanel`, xRange and yRange are tuples containing floats. The other two options are booleans indicating whether the user wants the respective range options chosen automatically by the graphing method. For the `TransformOptionsPanel`, the x and y transform options are strings indicating the type of transform method that should be applied to the data before graphing. Currently the only available options are `linear` and `log`. Linear amounts to not applying any transform to the data. The `log` transform can be applied by the particular matplotlib plot (if available), or using the FIND Transforms API, which will be discussed later in the section on Graphing Methods.

2.4.2 Graphing Methods

The second part of any Graphing plugin is the actual graph/plotting method that causes the underlying matplotlib library to draw to the plot Panel in the FIND user interface. The expected method signature and doc string are as follows:

```python
def graphingMethod(subplot, dims):
    """
    method-string-ID; method-name; Method description string
    """
```

The two parameters above are always passed to the graphing method, but do not necessarily need to be each used. The `dims` parameter is simply a list containing the currently selected data dimensions. The `subplot` parameter is a `Subplot` class instance. This class is the basic unit that represents a single graph/plot in the plot Panel in the UI. It contains the following attributes (lower case) and properties (capitalized) of use to plugin authors:

```
Subplot:
    Data
    Clustering
    opts
    axes
    parent
```

The `Data` and `Clustering` properties, respectively, provide the data array and (optionally) the array of cluster assignments. As stated previously, graphing methods must specify whether they apply to datasets, clusterings, or both. Thus, you only need to use what you specify as the target items. The `opts` attribute is a dictionary collected from the options dialog provided with the graphing method.

Important: Since the options dialog is only available to the user once the plot has been drawn, the `opts` attribute is empty the first time the graphing method is called. Thus, each graphing plugin must check whether the `opts` dict is empty and fill it with default options.

The `axes` attribute is an instance of the matplotlib Axes class. This class is the target for drawing, and all matplotlib provided plotting methods are available through it. The final `parent` attribute is an instance of the matplotlib Figure class. This class represents the entire drawing area in the UI, and is necessary for some applications such as providing scale bars and legends on plots (see the Heatmap2D plugin for an example).

As mentioned above, you may want to include data transformation options in your plugin. Currently the built-in options panel is not linked to the Transform methods module, and only allows for `linear` and `log` options. However, if you want to perform a log transformation before plotting (instead of asking matplotlib to do it as part of the plot), you can access the Transforms module with the following import:

```
import transforms.methods as tm
```

This module provides a `getMethod(strID)` method that returns the method specified by the string identifier of the transformation method/plugin. So to apply a log transform to your data with the built-in log method you might use the following line:

```
logData = tm.getMethod('log')(data)
```

The graphing method is not expected to return anything since the entire aim is to issue drawing commands to the underlying matplotlib engine.

Returning to the method declaration, the first line of the doc string must be semicolon-separated into three fields as seen above. The 'string-ID' field is what other plugin authors will give to the internal plot module in order to access the graphing method from within their own code. For example, an analysis plugin may want to display results in a temporary window that provides a simple Figure canvas. The 'method-name' is a short name that FIND will use for the menu item placed in Plugins>>Graph as well as the context plot menu that appears when clicking on a data item in the project tree. The final field will appear in the program status bar when a user moves the mouse over the menu item for the graphing plugin.

2.5 Input/Output Plugins

IO plugins are slightly different from the other plugin types. The `__all__` variable must still be populated with the registration method, but it must provide a string identifier and a reference to an `IOPlugin` (available from the pluginbase module) subclass. The base class provides structure for what is required of an IO plugin:

```
class IOPlugin(Plugin):
    """
    All IOPlugins are expected to provide methods for opening and/or saving
    data files.
    """
    def __init__(self, filename=None, fcData=None, window=None):
        self.filename = filename
        self.fcData = fcData
        self.window = window

    def register(self):
            """
            Returns a dictionary keyed on the FILE_INPUT and
            FILE_OUTPUT IDs (found in data.io) to indicate which (if any) methods
            provide input and output functionality.
            """
            pass

    def fileType(self):
        """
```

```
        Returns a string used for identifying the file type(s) this plugin is
        capable of reading/saving.

        ex: 'Comma Separated Values (*.csv)|*.csv'
        """
        pass

    def read(self):
        """
        Given the specified path of a data file, input the data and return
        it along with the column labels, and any annotations or analysis.

        :@rtype: tuple
        :@return: (labels, data, annotations, analysis)
        """
        pass

    def save(self):
        """
        Write the FC data to the specified file.
        """
        pass
```

As is specified above, the IOPlugin subclass must provide a second registration to the FIND plugin system, indicating whether it can read files, write files, or both. This is done by having the register method return a dictionary as specified above with the keys for reading and writing methods coming from:

```
from data.io import FILE_INPUT, FILE_OUTPUT
```

Thus, having the register method return a `dict` with only the `FILE_INPUT` key and the reading method will cause FIND to only use the class for file input (available as an option in File..Open). If the `FILE_OUTPUT` method is also specified, FIND will place a menu item in File..Export with the string identifier given in the module register method. The final parameter to the class initializer is `window`. This provides a reference to the FIND window class so that plugins can show dialog boxes (or other window subclasses) with options for reading or writing. For an example of this, see the CSV plugin.

2.6 Transformation Plugins

Data transformation plugins provide functionality to transform input data from one scale/space to another. The most basic example is a logarithmic transform which converts linear data to a logarithmic scale. The registration method for these plugins looks like the following:

```
def transform_register():
    return (transformMethod, MethodTransformScaleClass)
```

The `transformMethod` reference performs the actual transformation of user data. The `MethodTransformScaleClass` is an instance of the `ScaleBase` class found in the `matplotlib.scale` module. It is not required, so the register method can simply place `None` in the second slot of the tuple. However, if you wish to provide a plugin that is also automatically applicable to graphs/plots, then you will need to provide a subclass of `ScaleBase`. An example of creating your

own scale for plots is available at the matplotlib website. If you provide this class, FIND will automatically register your scale with the matplotlib engine, and it will be available to specify for any matplotlib plot that accepts scale requests.

The `transformMethod` method signature and doc string should look like the following:

```
def transformMethod(data, **kwargs):
    """
    string-ID; transformMethod name; Method description string
    """
    ...
    ...
    return transformed_data
```

The `data` parameter is (as with other plugins) an `m x n` array (numpy `ndarray`) with `m` data points (events), and `n` dimensions (channels). The `**kwargs` parameter is a dictionary containing options for use by the transform method. For example, FIND's built-in log transform accepts `base` and `min_clip` parameters indicating, respectively, the base of the log transform (2, 10, `e`) and the lower end the data should be clipped to when negative values are encountered (default: 10e-5).

Finally, as mentioned in the section on Graphing Plugins, the internal transforms package provides within its `methods` module, the means for any FIND code to use registered transforms. Specifically, the module provides a `getMethod(strID)` method that returns the method specified by the string identifier of the transformation method/plugin. So to apply a log transform to your data with the built-in log method you might use the following lines:

```
import transforms.methods as tm
logData = tm.getMethod('log')(data, base=10, min_clip=10e-8)
```

CHAPTER

THREE

INDICES AND TABLES

- *genindex*
- *modindex*
- *search*
